# Comparison of the Working Alliance in Blended Cognitive Behavioral Therapy and Treatment as Usual for Depression in Europe: Secondary Data Analysis of the E-COMPARED Randomized Controlled Trial

**DOI:** 10.2196/47515

**Published:** 2024-05-31

**Authors:** Asmae Doukani, Matteo Quartagno, Francesco Sera, Caroline Free, Ritsuko Kakuma, Heleen Riper, Annet Kleiboer, Arlinda Cerga-Pashoja, Anneke van Schaik, Cristina Botella, Thomas Berger, Karine Chevreul, Maria Matynia, Tobias Krieger, Jean-Baptiste Hazo, Stasja Draisma, Ingrid Titzler, Naira Topooco, Kim Mathiasen, Kristofer Vernmark, Antoine Urech, Anna Maj, Gerhard Andersson, Matthias Berking, Rosa María Baños, Ricardo Araya

**Affiliations:** 1 Department of Population Health London School of Hygiene & Tropical Medicine London United Kingdom; 2 Medical Research Council Clinical Trials Unit University College London London United Kingdom; 3 Department of Statistics Computer Science and Applications “G. Parenti” University of Florence Florance Italy; 4 Department of Psychiatry Amsterdam University Medial Centre, Vrije Universiteit Amsterdam Amsterdam Netherlands; 5 Department Clinical, Neuro, and Developmental Psychology Vrije Universiteit Amsterdam Amsterdam Netherlands; 6 Amsterdam Public Health Institute Amsterdam Netherlands; 7 Academic Department for Depressive Disorders Dutch Mental Health Care Amsterdam Netherlands; 8 Department of Basic Psychology, Clinical and Psychobiology Universitat Jaume I Castellón de la Plana Spain; 9 Centro de Investigación Biomédica en Red Fisiopatología Obesidad y Nutrición Instituto Carlos III Madrid Spain; 10 Department of Clinical Psychology and Psychotherapy University of Bern Bern Switzerland; 11 Unité de Recherche Clinique in Health Economics Assistance Publique–Hôpitaux de Paris Paris France; 12 Health Economics Research Unit Inserm University of Paris Paris France; 13 Faculty of Psychology SWPS University Warsaw Poland; 14 Department on Aging Netherlands Institute of Mental Health and Addiction (Trimbos Institute) Utrecht Netherlands; 15 Department of Clinical Psychology and Psychotherapy Friedrich-Alexander-Universität Erlangen-Nürnberg Erlangen Germany; 16 Department of Behavioural Sciences and Learning Linköping University Linköping Sweden; 17 Department of Clinical Medicine, Faculty of Health Sciences University of Southern Denmark Odense Denmark; 18 Centre for Digital Psychiatry Mental Health Services of Southern Denmark Odense Denmark; 19 Department of Neurology Inselspital Bern Bern University Hospital Bern Switzerland; 20 Department of Behavioral Sciences and Learning Linköping University Linköping Sweden; 21 Department of Clinical Neuroscience Karolinska Institute Stockholm Sweden; 22 Department of Biomedical and Clinical Sciences Linköping University Linköping Sweden; 23 Department of Clinical Psychology and Psychotherapy Friedrich-Alexander-University Erlangen-Nürnberg Erlangen Germany; 24 Department of Personality, Evaluation and Psychological Treatments Universidad de Valencia Valencia Spain; 25 Department of Health Service and Population Research Institute of Psychiatry, Psychology & Neuroscience King’s College London London United Kingdom

**Keywords:** blended psychotherapy, cognitive behavioral therapy, depression, digital mental health interventions, psychotherapy, mental health, program usability, therapeutic alliance, usability heuristics, working alliance

## Abstract

**Background:**

Increasing interest has centered on the psychotherapeutic working alliance as a means of understanding clinical change in digital mental health interventions in recent years. However, little is understood about how and to what extent a digital mental health program can have an impact on the working alliance and clinical outcomes in a blended (therapist plus digital program) cognitive behavioral therapy (bCBT) intervention for depression.

**Objective:**

This study aimed to test the difference in working alliance scores between bCBT and treatment as usual (TAU), examine the association between working alliance and depression severity scores in both arms, and test for an interaction between system usability and working alliance with regard to the association between working alliance and depression scores in bCBT at 3-month assessments.

**Methods:**

We conducted a secondary data analysis of the E-COMPARED (European Comparative Effectiveness Research on Blended Depression Treatment versus Treatment-as-usual) trial, which compared bCBT with TAU across 9 European countries. Data were collected in primary care and specialized services between April 2015 and December 2017. Eligible participants aged 18 years or older and diagnosed with major depressive disorder were randomized to either bCBT (n=476) or TAU (n=467). bCBT consisted of 6-20 sessions of bCBT (involving face-to-face sessions with a therapist and an internet-based program). TAU consisted of usual care for depression. The main outcomes were scores of the working alliance (Working Alliance Inventory-Short Revised–Client [WAI-SR-C]) and depressive symptoms (Patient Health Questionnaire-9 [PHQ-9]) at 3 months after randomization. Other variables included system usability scores (System Usability Scale-Client [SUS-C]) at 3 months and baseline demographic information. Data from baseline and 3-month assessments were analyzed using linear regression models that adjusted for a set of baseline variables.

**Results:**

Of the 945 included participants, 644 (68.2%) were female, and the mean age was 38.96 years (IQR 38). bCBT was associated with higher composite WAI-SR-C scores compared to TAU (*B*=5.67, 95% CI 4.48-6.86). There was an inverse association between WAI-SR-C and PHQ-9 in bCBT (*B*=−0.12, 95% CI −0.17 to −0.06) and TAU (*B*=−0.06, 95% CI −0.11 to −0.02), in which as WAI-SR-C scores increased, PHQ-9 scores decreased. Finally, there was a significant interaction between SUS-C and WAI-SR-C with regard to an inverse association between higher WAI-SR-C scores and lower PHQ-9 scores in bCBT (*b*=−0.030, 95% CI −0.05 to −0.01; *P*=.005).

**Conclusions:**

To our knowledge, this is the first study to show that bCBT may enhance the client working alliance when compared to evidence-based routine care for depression that services reported offering. The working alliance in bCBT was also associated with clinical improvements that appear to be enhanced by good program usability. Our findings add further weight to the view that the addition of internet-delivered CBT to face-to-face CBT may positively augment experiences of the working alliance.

**Trial Registration:**

ClinicalTrials.gov NCT02542891, https://clinicaltrials.gov/study/NCT02542891; German Clinical Trials Register DRKS00006866, https://drks.de/search/en/trial/DRKS00006866; Netherlands Trials Register NTR4962, https://www.onderzoekmetmensen.nl/en/trial/25452; ClinicalTrials.Gov NCT02389660, https://clinicaltrials.gov/study/NCT02389660; ClinicalTrials.gov NCT02361684, https://clinicaltrials.gov/study/NCT02361684; ClinicalTrials.gov NCT02449447, https://clinicaltrials.gov/study/NCT02449447; ClinicalTrials.gov NCT02410616, https://clinicaltrials.gov/study/NCT02410616; ISRCTN Registry ISRCTN12388725, https://www.isrctn.com/ISRCTN12388725?q=ISRCTN12388725&filters=&sort=&offset=1&totalResults=1&page=1&pageSize=10; ClinicalTrials.gov NCT02796573, https://classic.clinicaltrials.gov/ct2/show/NCT02796573

**International Registered Report Identifier (IRRID):**

RR2-10.1186/s13063-016-1511-1

## Introduction

### Background

Depression is one of the most significant contributors to the global disease burden, affecting an estimated 264 million people globally [1,2]. Depression accounts for 7.2% of the overall disease burden in Europe, costing an estimated €113,405 billion (US $123,038 billion) per year. However, 45% of people with major depression will go untreated [3]. High costs and suboptimal access to mental health care are among the many reasons to foster digital mental health interventions (DMHIs), which promise greater quality of care and lower costs of delivery [4,5].

Evidence concerning the effectiveness of DMHIs has increased substantially over the past decade. Growing evidence indicates that internet-delivered cognitive behavioral therapy (iCBT) might be just as effective as face-to-face cognitive behavioral therapy (CBT) for a range of mental health conditions, particularly depression [6-13]. iCBT is delivered with varying degrees of support ranging from a stand-alone self-administered digital program to a blended treatment with the active involvement of a therapist through regular face-to-face meetings. Blended psychotherapies provide higher levels of therapist support compared to guided approaches that provide minimal or some guidance from a mental health practitioner [4]. Blended delivery has gained interest, with emerging evidence suggesting that such interventions can lead to improved adherence and treatment outcomes [14].

As interest in DMHIs increases, considerable attention has centered around the concept of the client-therapist alliance, of which there are many variations (therapeutic, working, helping, etc). While different therapeutic approaches have historically failed to agree on a definition of the alliance, Edward Bordin [15-17] proposed a pan-theoretical tripartite conceptualization called the working alliance that is characterized by 3 key dimensions, including the emotional “bond” between the client and the therapist, the agreement on the therapeutic “goals,” and the “task” needed to advance the client’s goals toward clinical improvement. This concept is particularly important because it has consistently predicted positive treatment outcomes for a range of psychological approaches, including CBT for depression [18-20].

The client-therapist alliance was identified as a key research priority for research policy and funding in digital technologies in mental health care, in a large consensus study involving people with lived experiences of mental health problems and service use, their carers, and mental health practitioners [21]. The integration of digital technologies in psychotherapy has led to changes in the way the alliance is conceptualized and assessed [19], with variability depending on the type of DMHI (digital program [22], avatar [23], or mobile app [24]).

### Prior Work

The literature investigating the client-therapist alliance has largely focused on addressing 2 key questions. The first question is “Do *alliance* scores predict changes in clinical outcomes?” [21,25-29], and the second question, which has been focused on to a lesser extent, is “Does the alliance vary depending on how psychotherapy is delivered?” Systematic reviews that have addressed these questions specifically in relation to interventions that are guided, adopt CBT [21], or target the treatment of depression [27] found that the working alliance can be established in guided DMHIs at a comparable level to face-to-face therapy [21]; however, the literature on the outcome-alliance relationship is mixed [21,26,27].

To this end, only 3 studies have examined the working alliance in blended CBT (bCBT). The first was an uncontrolled study in Sweden, which offered 4 face-to-face and 10 iCBT sessions to a total of 73 participants in primary care services and which was part of the E-COMPARED (European Comparative Effectiveness Research on Blended Depression Treatment versus Treatment-as-usual) study [30]. The findings showed that the alliance was rated highly by both clients and therapists. However, only therapist alliance ratings were associated with client score changes in depression, while client ratings were not.

The second study was conducted in the Netherlands and recruited 102 participants from specialist care services. Participants were either randomized to bCBT (n=47), which consisted of a 20-week intervention (10 face-to-face and 10 online sessions), or a control condition (n=45), which consisted of 15-20 face-to-face CBT sessions [31]. Similar to the findings from the study conducted in Sweden [30], the working alliance was rated highly by both clients and therapists, and no differences were observed between scores. Client ratings of the working alliance were associated with lower depression scores over time in face-to-face CBT but not in bCBT. Therapist working alliance ratings were not significantly associated with depression scores over time in both treatment conditions [31].

The third and most recent study was conducted in Denmark. The study recruited a total of 76 participants who were either randomized to bCBT (n=38), which consisted of 6 face-to-face sessions that were alternated with 6-8 online modules of an internet-based program, or a control condition (n=38), which consisted of 12 face-to-face CBT sessions [32]. The findings showed a significant difference in client and therapist working alliance scores, in which clients rated their working alliance higher than therapists. However, only the therapist ratings across conditions were significantly associated with outcomes in depression. Working alliance ratings across face-to-face CBT and bCBT were comparable. Working alliance ratings in both face-to-face CBT and bCBT did not significantly predict treatment outcomes. It is not clear why an in-group effect was found for therapists across the pooled data and not within treatment conditions [32]. These findings might indicate that the study was not powered enough to detect an association for client ratings in each treatment condition.

While research has mainly focused on measuring the alliance between the client and therapist, emerging qualitative research suggests that DMHIs may offer additional relational alliance benefits [29,33,34]. An example comes from a qualitative study that examined the working alliance demands in a bCBT intervention for people with mild-to-moderate depression in the United Kingdom, as part of the E-COMPARED trial [35]. Qualitative data indicated a potential fourth dimension called “usability heuristics,” which appeared to uniquely promote the working alliance in bCBT. Usability heuristics defines the digital program’s role in promoting active engagement, self-discovery, and autonomous problem-solving, with higher levels expected to enhance the quality of the working alliance. Features that enable “usability heuristics” include digital technologies that increase access and immediacy to the therapeutic task (availability), appropriately respond to the client’s input (interactivity), are easy to use, have esthetic appeal, and promote self-directed therapy [36]. Findings regarding usability heuristics and the respective subfeatures were also reported in another qualitative study that tested this framework in a Spanish sample of participants who experienced self-guided or low-intensity supported iCBT [37]. It is therefore possible that experiences of digital program features may influence the way that the working alliance is experienced in blended formats of CBT [36].

### Aims and Objectives

To our knowledge, we report the largest investigation of the working alliance in bCBT for depression, using pooled data from 9 country sites involved in a pragmatic noninferiority randomized controlled trial investigating the effectiveness of bCBT for depression when compared with treatment as usual (TAU) [35]. Further to this, our study will explore if system usability, a newly conceptualized feature of the working alliance, in bCBT interacts with the working alliance and treatment outcome association [36]. Our primary objectives are to test the difference in working alliance scores between bCBT and TAU (objective 1), and determine if working alliance scores are associated with depression scores (objective 2). Our secondary objective is to test for an interaction between system usability and the working alliance with regard to an association between the working alliance and depression scores in bCBT (objective 3).

## Methods

### Study Design and Settings

We conducted a nonprespecified secondary analysis of data collected in the E-COMPARED study, a large European 2-arm, noninferiority randomized controlled trial investigating the effectiveness of bCBT compared with TAU across 9 European countries (France: ClinicalTrials.gov NCT02542891, September 4, 2015; Germany: German Clinical Trials Register DRKS00006866, December 2, 2014; The Netherlands: Netherlands Trials Register NTR4962, January 5, 2015; Poland: ClinicalTrials.gov NCT02389660, February 18, 2015; Spain: ClinicalTrials.gov NCT02361684, January 8, 2015; Sweden: ClinicalTrials.gov NCT02449447, March 30, 2015; Switzerland: ClinicalTrials.gov NCT02410616, April 2, 2015; United Kingdom: ISRCTN Registry ISRCTN12388725, March 20, 2015; Denmark: ClinicalTrials.gov NCT02796573, June 1, 2016) [35,38]. Data were collected between April 2015 and December 2017. Clients seeking treatment for depression were recruited, assessed, and treated across routine primary care in Germany, Poland, Spain, Sweden, and the United Kingdom, and specialized mental health services in France, the Netherlands, Switzerland, and Denmark [35]. Following the start of recruitment, an additional satellite site was added in Denmark to boost recruitment [38]. The E-COMPARED trial was funded by the European Commission FP7-Health-2013-Innovation-1 program (grant agreement number: 603098).

### Participants

Recruitment procedures differed in each country, but all sites screened new clients seeking help for depression, who scored 5 or higher on the Patient Health Questionnaire-9 (PHQ-9) [39]. The study was explained to potential participants either face-to-face or over a telephone call. Clients who agreed to take part in the study were invited to an initial appointment to assess eligibility. The inclusion criteria applied at all sites were as follows: age ≥18 years and meeting the diagnostic criteria for major depressive disorder as confirmed by the MINI International Neuropsychiatric Interview (M.I.N.I) version 5.0 [40]. The exclusion criteria were as follows: high risk of suicide and psychiatric comorbidity (ie, substance dependence, bipolar affective disorder, psychotic illness, or obsessive compulsive disorder) assessed during the M.I.N.I. interview; receiving psychological treatment for depression in primary or specialized mental health care at the point of recruitment; inability to comprehend the spoken and written language of the country site; lacking access to a computer or a fast internet connection (ie, broadband or comparable); and lacking a smartphone or being unwilling to carry a smartphone if one was provided by the research team [35].

After baseline assessments, participants were randomized to 1 of 2 treatment arms (bCBT or TAU) using block randomization, with stratification by country [35]. All participants provided written informed consent before taking part in the trial [35].

### Ethical Considerations

The trial was conducted in accordance with the Declaration of Helsinki and was approved by all local ethics committees. Ethics approval to conduct a secondary analysis was obtained from the London School of Hygiene and Tropical Medicine Research Ethics Committee on October 7, 2019 (ethics reference number: 17852). For further information on the trial, including local ethics approvals and the randomization process, see the trial protocol [35].

### Interventions: bCBT and TAU

bCBT for depression consisted of integrating a digital program (iCBT plus mobile app) with face-to-face CBT in a single treatment protocol [35,41]. iCBT programs included 4 mandatory core modules of CBT (ie, psychoeducation, behavioral activation, cognitive restructuring, and relapse prevention) plus optional modules (eg, physical exercise and problem solving) typically completed at home, while face-to-face CBT was delivered in the clinic [35]. Clients worked through treatment modules, completed exercises, and monitored their symptoms on the digital program, while face-to-face sessions were used by the therapist to set up modules, monitor client progress, and address client-specific needs. Sequencing and time spent on each module were flexibly applied; however, the 4 mandatory modules on the digital program had to be completed. Data on treatment and dosage were not collected for TAU in the trial. See Table 1 for a breakdown of recruitment, bCBT format and dosage, and treatments offered in TAU across all country sites [30,35,42]. It was not possible to blind therapists to treatment allocation; however, assessors were blinded [35].

**Table 1:** Overview of recruitment in the trial, blended cognitive behavioral therapy format and dosage, and treatment offered in the treatment as usual arm by country site.

Country	Recruitment		bCBT^a^ format and dosage					TAU^b^ allocation
	Treatment setting	Recruitment procedure	Platform	Duration (weeks)	Online/face-to face, n	Sequencing^c^		
France	Specialized mental health care	New or regular patients recruited by CBT^d^ therapists from 11 expert centers throughout France.	Moodbuster	16	8/8	Alternate	Face-to-face CBT	
Germany	Primary care	Recruitment in the waiting room of GP^e^ clinics or during GP consultations.	Moodbuster	11-13	10/6	Alternate	GP care (eg, watchful waiting, medication prescription, referral to medical specialists, or face-to-face CBT)	
Netherlands	Specialized mental health care	Recruitment through mood disorder departments of 3 outpatient clinics in Amsterdam and Leiden.	Moodbuster	20	10/10	Alternate	Evidence-based face-to-face psychotherapy (mainly CBT, interpersonal psychotherapy, problem-solving therapy, antidepressant medication, or a combination of these).	
Poland	Primary care	Recruitment through primary care centers by CBT therapists (licensed and in training) in 5 major cities in Poland (Warsaw, Sopot, Poznan, Katowice, and Wroclaw).	Moodbuster	6-10	6/7	Alternate	Face-to-face CBT	
Spain	Primary care	Recruitment through routine primary care from the Spanish National Health System in several cites (Valencia, Castellón, and Zamora).	Smiling is fun	10	8/3	1-4-1-4-2	Prescribed medication by the GP or received face-to-face CBT and interpersonal psychotherapy or supportive therapy once a month	
Sweden	Primary care	Recruitment through collaborating primary care clinics in 3 Swedish counties (Stockholm, Linköping, and Västerås). Posters and leaflets were distributed in the waiting areas or were provided to GPs in clinics, who in turn referred potentially eligible participants.	Iterapi	10	6/4	Alternate	Usual care paths in Sweden, including general practitioner care; eg, watchful waiting, medication prescription, referral to medical specialist, or face-to-face CBT	
Switzerland	Specialized mental health care	Recruitment through 2 outpatient clinics (Bern and Zurich) and individual therapists.	Deprexis	18	9/9	Alternate	Face-to-face CBT	
United Kingdom	Primary care	Recruitment through the IAPT^f^ NHS^g^ program across London, Norfolk, Suffolk, and Berkshire. The IAPT program primarily provides evidence-based psychological therapies to people with depression and anxiety disorders.	Moodbuster	11	5/6	Alternate	Face-to-face CBT	
Denmark^h^	Specialized mental health care	Recruitment through the Center for Telepsychiatry in specialized mental health care at the Mental Health Services of the Region of Southern Denmark, where patients are referred to the study by clinicians. Initially, patients are self-referred to the Center for Telepsychiatry.	NoDep	12	6-8/6	Alternate	Face-to-face CBT	

^a^bCBT: blended cognitive behavioral therapy.

^b^TAU: treatment as usual.

^c^Sequencing of face-to-face and online sessions can include more than one session per week for either component.

^d^CBT: cognitive behavioral therapy.

^e^GP: general practitioner.

^f^IAPT: improving access to psychological therapy.

^g^NHS: National Health Service.

^h^Denmark was added as a satellite recruitment site [38] after the commencement of the project.

Based on the registered data, 194 therapists delivered trial interventions. In Germany, therapists only delivered bCBT in the treatment arm, whereas therapists from the remaining 8 country sites delivered interventions across both treatment arms. The risk of contamination was not perceived as a concern, as CBT was also offered in TAU, with the focus of the trial on investigating the blending of an internet-based intervention with face-to-face CBT when compared to routine care. Data on therapist ratings of the working alliance will be published in a separate paper to enable comprehensive reporting and discussion of the findings.

### Measures

#### Diagnostic Assessment

In the E-COMPARED study [35], a diagnosis of major depression according to the Diagnostic and Statistical Manual of Mental Disorders IV (DSM-IV) was established at baseline using the M.I.N.I [40], a structured diagnostic interview that has been translated into 65 languages and is used for both clinical and research practice. The interview compares well with the Structured Clinical Interview for DSM-IV disorders [43] and the Composite International Diagnostic Interview [40,43]. The M.I.N.I. was also used to assess the following comorbid disorders that were part of the exclusion criteria: substance dependence, bipolar affective disorder, psychotic illness, and obsessive-compulsive disorder. The M.I.N.I was administered face-to-face or via telephone at baseline and 12-month follow-up assessments. Telephone administration of diagnostic interviews has shown good validity and reliability [44,45].

#### Primary Measures

The study outcomes were the working alliance and depression severity, which were measured using the Working Alliance Inventory-Short Revised–Client (WAI-SR-C) [46] and the PHQ-9 [39], respectively. The WAI-SR-C scale is based on the theory of working alliance containing 3-item subscales assessing bond, task, and goals by Bordin [15,16]. The 12 items are rated on a 5-point scale from 1 (seldom) to 5 (always), with total scores ranging between 12 and 60. Higher scores on the scale indicate better working alliance. The WAI-SR-C scale has demonstrated good reliability (internal consistency) for all 3 factors, including the bond, task, and goals subscales (Cronbach α=0.92, 0.92, and 0.89, respectively) [47]. The scale has been shown to be correlated with other therapeutic alliance scales such as the California Therapeutic Alliance Rating System [19,48] and the Helping Alliance Questionnaire-II [19,49]. The WAI-SR-C scale was only administered at 3-month assessments. Data for the WAI-SR-C scale were not collected in the TAU arm of the Swedish country site.

The PHQ-9 [39] was used to assess depression as the trial’s primary clinical outcome. The PHQ-9 is a 9-item scale that can be used to screen and diagnose people for depressive disorders. Each of the 9 items is scored on a 4-point scale from 0 (not at all) to 3 (nearly every day). The total score ranges between 0 and 27, with higher scores indicating greater symptom severity. Depression severity can be grouped into the following: mild (score 0-5), moderate (6-10), moderately severe (11-15), and severe (≥16). The PHQ-9 has been shown to have good psychometric properties [39] and has demonstrated its utility as a valid diagnostic tool [50]. The PHQ-9 was administered at the baseline and 3-, 6-, and 12-month assessments; however, this study only used baseline and 3-month assessment data as the study was interested in investigating depression scores that generally corresponded to before and after treatment.

#### Other Measures

System Usability Scale-Client (SUS-C) [51,52] was used to assess the usability of the digital programs. The SUS-C is a 10-item self-reported questionnaire. Items are measured on a 5-point scale ranging from 1 (strongly disagree) to 5 (strongly agree). The total SUS-C score ranges between 10 and 50 to produce a global score. Higher scores indicate better system usability. The total sum score has been found to be a valid and interpretable measure to assess the usability of internet-based interventions by professionals in mental health care settings [53]. The SUS has shown high internal reliability (eg, coefficient Ω=0.91) and good concurrent validity and sensitivity [52,53]. The SUS-C was administered at the 3-month follow-up assessment.

Demographic data on the participant’s gender, age, educational attainment, marital status, and country site were collected at baseline. Baseline variables entered as covariates in the regression models included age, gender (male, female, and other), marital status (single, divorced, widowed, living together, and married), and educational level (low, middle, and high, corresponding to secondary school education or equivalent [low], college or equivalent [middle], and university degree or higher [high]).

Baseline data were completed online, face-to-face, via telephone, or a combination of these approaches. The 3-month follow-up assessments were largely completed online, with the exception of the PHQ-9 that was collected via telephone to maximize data collection of the trial’s primary outcome. Data that were directly collected by researchers (ie, either in person or via telephone) were double entered to increase the accuracy of the data entry process.

### Statistical Analysis

The study used an intention-to-treat (ITT) population for the data analysis [54]. While the ITT approach is standard for RCTs, some methodologists advise that a per-protocol population is more suitable for pragmatic noninferiority trials owing to concerns that a “flawed trial” is likely to incorrectly demonstrate noninferiority (eg, a trial that loses the ability to distinguish any true differences between treatment groups that are present). However, contrary to the primary analysis in the E-COMPARED trial, noninferiority tests were not performed in our analyses. A decision was made to use a pure ITT population in order to maintain the original treatment group composition achieved after the random allocation of trial participants, therefore minimizing the confounding between the treatment groups and providing unbiased estimates of the treatment effects on the working alliance [54].

Data of the E-COMPARED trial were downloaded from a data repository. All analyses employed an ITT population. All models were adjusted for baseline PHQ-9 scores, age, gender, marital status, educational attainment, and country site. Analyses were performed on SPSS (version 26 or above) [55], STATA (version 16 or above) [56], and PROCESS Macro plug-in for SPSS (version 3.5 or above) [57]. Reported *P* values are 2-tailed, with significance levels at *P*≤.05.

#### Treatment Assignment as a Predictor for WAI-SR-C Scores at 3-Month Assessments

In order to test if treatment assignment predicted WAI-SR-C scores at 3-month assessments (objective 1), a fixed effects linear regression model [58] was fitted separately for WAI-SR-C composite and subscale scores (goals, task, and bond). Four models were fitted altogether.

#### Association Between PHQ-9 Scores and WAI-SR-C Scores at 3-Month Assessments

To determine if WAI-SR-C scores were associated with PHQ-9 scores at 3-month assessments (objective 2), a fixed effects linear regression model was fitted to investigate this association separately for the bCBT and TAU arms in order to understand the alliance-outcome association within different treatment conditions in the trial. The model was also fitted separately for WAI-SR-C composite and subscale scores. Eight models were fitted altogether. 

#### Testing the Interaction Between WAI-SR-C and SUS-C Scores With Regard to the Relationship Between WAI-SR-C and PHQ-9 Scores

To test the interaction between 3-month SUS-C and 3-month WAI-SR-C scores in a model examining the relationship between 3-month WAI-SR-C and 3-month PHQ-9 scores, a multiple regression model was fitted separately for WAI-SR-C composite and subscale scores in order to estimate the size of the interaction. Four models were fitted altogether.

#### Missing Data

Multiple imputation was used to handle high levels of missing data, under the missing at random (MAR) assumption. In particular, 36.6% (345/943) of data were missing for the PHQ-9, 20.7% (195/943) were missing for the WAI-SR-C, and 27.9% (133/476) were missing for the SUS-C at 3-month assessments. We imputed data sets using the chained equation approach [59]. Tabulations of missing data across treatment conditions and country sites are presented in Tables S1-S3 in Multimedia Appendix 1. Chi-square results of differences in missing and complete data between E-COMPARED country sites are presented in Tables S4 and S5 in Multimedia Appendix 1. In the imputation model, we included all variables that were part of the analyses, including observations from the PHQ-9 at baseline and demographic variables. To account for the interaction term in the regression model, data were imputed using the just another variable (JAV) approach [60]. Multiple imputation was performed separately for bCBT and TAU to allow for condition-specific variables to be considered. For example, the SUS-C variable was only entered in the bCBT arm, as those in the TAU arm did not use a digital program.

#### Post hoc Analysis

Post hoc sensitivity analyses were conducted to examine if the multiple imputation approach that was used to handle missing data would lead to different conclusions when compared to a complete case analysis. Under the MAR assumption, consistent findings between the primary analysis and sensitivity analysis can strengthen the reliability of the findings [61-64], at least in situations where both the primary and sensitivity analyses are expected to be valid under similar assumptions (eg, multiple imputation and complete case analysis under the MAR assumption in the outcome variable only).

Owing to the heterogeneity of interventions offered in the TAU arm within the current pragmatic trial, a subgroup analysis was conducted to explore the magnitude of treatment effects on the working alliance when using a subset of the sample, which compared bCBT with face-to-face CBT offered in the TAU arm in Denmark, France, Poland, Switzerland, and the United Kingdom country sites of the E-COMPARED trial [35]. The subanalysis replicated the main analysis in just 5 country sites. This enabled the working alliance in bCBT to be directly compared with a defined comparator. Results between the primary analysis and the subanalysis were compared to understand if results vary when there are multiple interventions in TAU and when there is a defined comparator (ie, face-to-face CBT) [65-67].

## Results

### Clinical and Demographic Characteristics

Table 2 summarizes the baseline characteristics. Among the 943 participants who consented and were randomized in the trial (bCBT=476; TAU=467) (See Figure S1 in Multimedia Appendix 1 for the trial’s profile), most were female (644/943, 68.3%), were middle-aged, and had a university degree or higher (447/943, 47.4). The PHQ-9 scores (median 15, IQR 7) reflected depression of moderate severity. PHQ-9 scores at 3 months will be reported in the main trial paper, which is being prepared. The median WAI-SR-C score was 47.42 (IQR 6) in the bCBT arm and 42 (IQR 8) in the TAU arm. The median SUS-C score was 42 (IQR 9) in the bCBT arm. See Table 3 for the median (IQR) values of the WAI-SR-C and SUS-C scores across treatment groups, and see Tables S6-S8 in Multimedia Appendix 1 for the median (IQR) values of the WAI-SR-C and SUS-C scores by country site.

**Table 2:** Baseline characteristics of participants.

Characteristic		bCBT^a^ (n=476)	TAU^b^ (n*=*467)	Total (N=943)
**Age (years)**				
	Median (IQR)	38 (22)	37 (22)	38 (23)
	Range (minimum-maximum)	18-74	18-78	18-78
Gender (female), n (%)		318 (67)	326 (70)	644 (68)
**Marital status, n (%)**				
	Single	159 (33)	155 (33)	314 (33)
	Divorced	60 (13)	43 (9)	103 (11)
	Widowed	3 (1)	6 (1)	9 (1)
	Living together	95 (20)	111 (24)	206 (22)
	Married	159 (33)	152 (33)	311 (33)
**Level of education^c^, n (%)**				
	Secondary school, equivalent	72 (15)	74 (16)	146 (16)
	College, equivalent	179 (38)	170 (36)	349 (37)
	University degree or higher	225 (47)	222 (48)	447 (47)
**Country site (n=943)^d^, n (%)**				
	Germany	86 (18)	87 (19)	173 (18)
	Sweden	73 (15)	68 (15)	141 (15)
	Netherlands	53 (11)	49 (11)	102 (11)
	United Kingdom	49 (10)	52 (11)	101 (11)
	Spain	64 (13)	63 (14)	127 (14)
	France	51 (11)	54 (12)	105 (11)
	Switzerland	26 (6)	24 (5)	50 (5)
	Poland	42 (9)	42 (9)	84 (9)
	Denmark	32 (7)	28 (6)	60 (6)
**Baseline PHQ-9^e^ scores^f^**				
	Median (IQR)	15 (7)	16 (6)	15 (7)
	Range (minimum-maximum)	4-27	5-26	4-27

^a^bCBT: blended cognitive behavioral therapy.

^b^TAU: treatment as usual.

^c^Data collected were in respect to what would be considered low, middle, and high levels of education in each setting. Data were missing for 1 of 943 (0.2%) individuals in the bCBT arm.

^d^Self-reported country of birth can be found in Table S9 in Multimedia Appendix 1.

^e^PHQ-9: Patient Health Questionnaire-9.

^f^PHQ-9 severity cutoff points are as follows: 5-9, mild depression; 1-14, moderate depression; 15-19, moderately severe depression; and ≥20, severe depression [39].

**Table 3:** Data of Working Alliance Inventory-Short Revised–Client composite and subscale scores and System Usability Scale-Client scores collected at 3-month follow-up assessments.

Scale		bCBT^a^ (n=476)	TAU^b^ (n=467)	Total (N=943)
**WAI-SR-C^c^, median (IQR)**				
	Composite	47.42 (6)	42 (8)	46 (9.2)
	Goals	16.08 (3.4)	14 (3.9)	15.50 (4.7)
	Task	14.45 (3)	12.83 (4)	14 (4)
	Bond	17 (4)	15.43 (3)	16 (3.7)
SUS-C^d^, median (IQR)		42 (9)	N/A^e^	N/A

^a^bCBT: blended cognitive behavioral therapy.

^b^TAU: treatment as usual.

^c^WAI-SR-C: Working Alliance Inventory-Short Revised–Client.

^d^SUS-C: System Usability Scale-Client.

^e^N/A: not applicable.

### Treatment Assignment as a Predictor for WAI-SR-C Scores

Treatment assignment significantly predicted WAI-SR-C composite, goals, task, and bond scores (See Table 4 for model summaries). Being allocated to bCBT predicted higher WAI-SR-C composite and subscale scores at 3-month assessments when compared to TAU.

**Table 4:** Adjusted linear regression models of treatment assignment as a predictor for Working Alliance Inventory-Short Revised–Client composite and subscale (goals, task, and bond) scores.

WAI-SR-C^a^ (outcome)^b^	*B*^c^ (95% CI)	*P* value
Composite	5.67 (4.48-6.86)	<.001
Goals	2.32 (1.87-2.78)	<.001
Task	1.99 (1.53-2.44)	<.001
Bond	1.36 (0.91-1.81)	<.001

^a^WAI-SR-C: Working Alliance Inventory-Short Revised–Client.

^b^Separate models were generated for WAI-SR-C composite and subscale scores (ie, goals, task, and bond).

^c^Unstandardized beta.

### Association Between PHQ-9 Scores and WAI-SR-C Scores at 3-Month Assessments

Across both treatment arms, WAI-SR-C composite scores and goals and task subscale scores were significantly associated with PHQ-9 scores, in which lower PHQ-9 scores were associated with higher WAI-SR-C composite scores and goals and task subscale scores. WAI-SR-C bond scores were not significantly associated with PHQ-9 scores in both treatment arms (see Table 5 for model summaries).

**Table 5:** Adjusted linear regression models of associations between Patient Health Questionnaire-9 and Working Alliance Inventory-Short Revised–Client composite and subscale (goals, task, and bond) scores at 3-month assessments.

WAI-SR-C^a^ (outcome)^b^	bCBT^c^			TAU^d^	
	*B*^e^ (95% CI)	*P* value	*B*^e^ (95% CI)		*P* value
Composite	−0.12 (−0.17 to −0.06)	<.001	−0.06 (−0.11 to −0.02)		.01
Goals	−0.26 (−0.41 to −0.11)	.001	−0.13 (−0.25 to −0.00)		.04
Task	−0.38 (−0.52 to −0.24)	<.001	−0.18 (−0.32 to −0.05)		.008
Bond	−0.13 (−0.27 to 0.02)	.10	−0.12 (−0.25 to 0.01)		.07

^a^WAI-SR-C: Working Alliance Inventory-Short Revised–Client.

^b^Separate models were generated for WAI-SR-C composite and subscale scores (ie, goals, task, and bond).

^c^bCBT: blended cognitive behavioral therapy.

^d^TAU: treatment as usual.

^e^Unstandardized beta.

### Testing the Interaction Between WAI-SR-C and SUS-C Scores With Regard to the Relationship Between WAI-SR-C and PHQ-9 Scores

**Figure 1:**
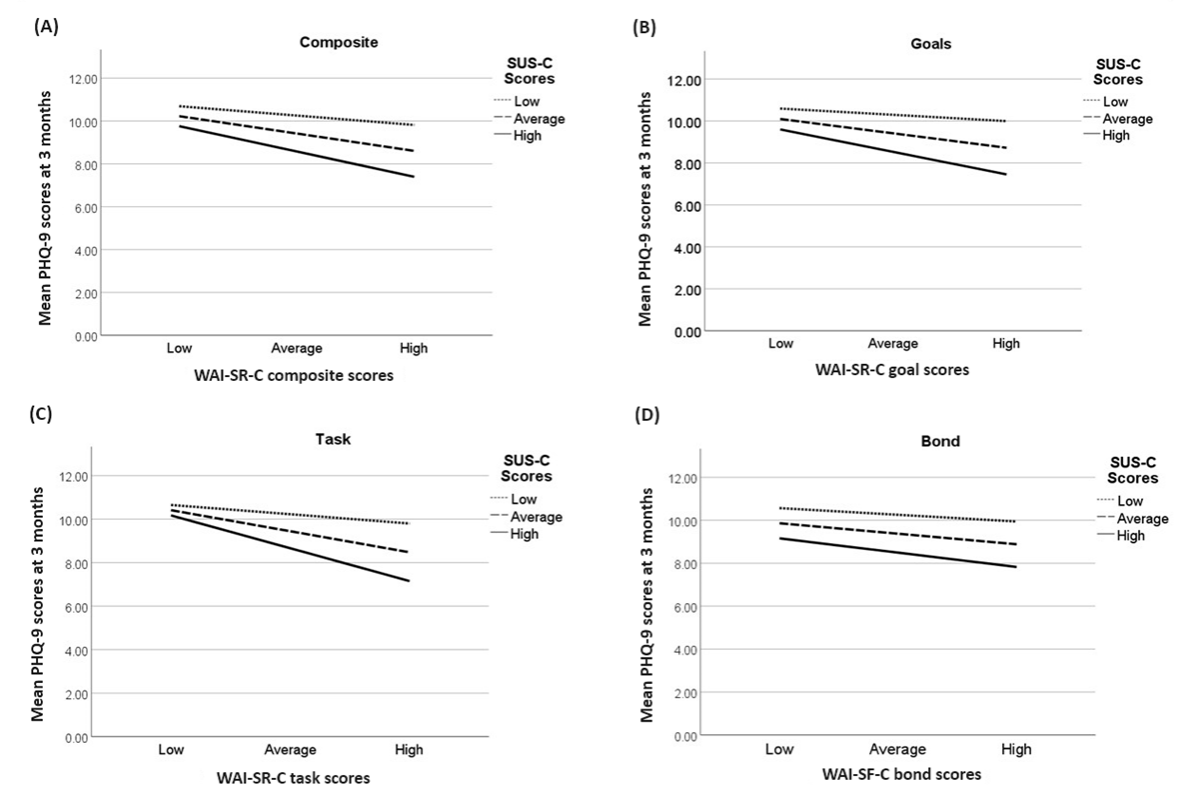
Multiple line graphs of the interaction between SUS-C and WAI-SR-C with regard to the association between WAI-SR-C (composite scores and goals, task, and bond subscale scores) and PHQ-9 scores at 3-month assessments in the cognitive behavioral therapy arm. PHQ-9: Patient Health Questionnaire-9; SUS-C: System Usability Scale-Client; WAI-SR-C: Working Alliance Inventory-Short Revised–Client.

There was a significant interaction between WAI-SR-C and SUS-C scores with regard to the association between WAI-SR-C composite scores and PHQ-9 scores at 3 months (*b*=−0.008, 95% CI −0.01 to −0.00; *P*=.03). Similar findings were noted for the goals (*b*=−0.021, 95% CI −0.04 to −0.00; *P*=.03) and task (*b*=−0.028, 95% CI −0.05 to −0.01; *P*=.003) subscales but not for the bond subscale (*b*=−0.010, 95% CI −0.03 to 0.01; *P*=.30). Figure 1 shows the presence of an inverse association between composite WAI-SR-C (for composite, and the goals and task subscales but not the bond subscale) and PHQ-9 scores among those with high SUS-C scores.

### Sensitivity and Subgroup Results

The sensitivity analysis with the complete case data set and subgroup analysis of 5 country sites that only offered face-to-face CBT in the TAU arm produced results that were comparable to those reported in the main paper. However, the interaction between SUS-C (and all subscales) and WAI-SR-C scores with regard to the association between WAI-SR-C and PHQ-9 scores was not significant in terms of sensitivity. Other differences are summarized in Results S2 in Multimedia Appendix 1, while the full results of the sensitivity and subgroup analyses can be found in Results S3 and S4 in Multimedia Appendix 1.

## Discussion

### Principal Findings

This study investigated the client-rated working alliance in a bCBT intervention for depression when compared to TAU [35]. Overall, our study found that treatment allocation (bCBT versus TAU) was a significant predictor of working alliance scores, in which ratings of the working alliance (composite scale and goals, task, and bond subscales) were higher in bCBT than in TAU. The working alliance was significantly associated with treatment outcomes. Across both bCBT and TAU groups, as working alliance scores increased, PHQ-9 scores decreased for composite, goals, and task scores but not for bond scores. Finally, there was a significant interaction between average and above-average system usability and higher working alliance (composite scale and goals and task subscales, but not bond subscale) scores when examining the relationship between the working alliance and PHQ-9 scores at 3-month assessments.

To our knowledge, our study is the first to report that working alliance composite scores and all subscale scores were higher in bCBT than in TAU. A post hoc analysis using data from country sites that only offered face-to-face CBT in the TAU arm found that the working alliance was significantly higher in the bCBT arm compared to face-to-face CBT. These findings indicate that a blended approach may offer additional alliance-building benefits when compared to face-to-face CBT and other types of usual care for depression offered in TAU such as talking therapies and psychopharmacological interventions. A possible explanation for our findings is that the digital elements of the intervention may enable better definition and coverage of the goals and the task than what might be possible in face-to-face sessions alone [68]. A study exploring program usage across 4 country sites of the E-COMPARED study found that clients received an average of 10 messages from their therapists online [69]. Features of the digital program that enabled the client to receive contact from the therapist away from the clinic may therefore play a role in increasing the availability of the therapist and enhancing opportunities to further strengthen the working alliance [69].

Further support for our findings comes from a qualitative study that examined the working alliance in bCBT in the United Kingdom country site of the E-COMPARED trial [36], which found that participants preferred bCBT compared to face-to-face CBT alone. The “immediacy” of access to the therapeutic task was reported to enhance engagement with the intervention and provide a higher sense of control and independence. The digital program was also described as a “secure base” that allowed participants to progressively explore self-directed treatment [36]. Similarly, a qualitative study from the German country site of the E-COMPARED trial found that bCBT was perceived to strengthen patient self-management and autonomy in relation to place and location [70].

Our study appears to be the first to identify a significant association between lower depression scores and higher working alliance composite scores and goals and task subscale scores but not bond subscale scores. In alignment with our findings, a narrative review of the working alliance in online therapy found that most guided iCBT studies included in the review reported significant associations between outcomes and the task and goals subscale scores but not the bond subscale scores [26]. A possible explanation could be that the bond is experienced differently in bCBT compared to traditional formats of CBT [26]. Bordin’s [15,16] conceptualization of the working alliance suggests that while the pan-theoretical theory allows for the basic measurement of the goals, task, and bond to produce beneficial therapeutic change, the ideal alliance profile is likely to be different across therapeutic approaches and interventions [15,16,18]. The findings may therefore indicate that the working alliance profile might differ in b-CBT. However, further research is needed to investigate this.

Finally, our finding that average and higher system usability ratings may strengthen the working alliance (especially the task subscale) may point to the digital programs’ influence on how the working alliance is experienced. This is not surprising given that CBT activities (eg, content and exercises) were primarily completed in the iCBT program and may indicate its relevance in the building of the working alliance and in supporting the task within a capacity that is potentially parallel to the bond. These findings partially test and support a conceptual framework of the working alliance that incorporates features that are derived from the digital program within a blended setting called “digital heuristics” (the promotion of active engagement and autonomous problem solving) in which “ease of use” and “interactivity” were identified as key features for optimizing “active engagement” with the task in the iCBT program [36]. These qualitative findings were mirrored in another study that tested the abovementioned framework, in which digital heuristics emerged as a fourth dimension when examining the working alliance in self-guided and low-intensity supported iCBT for depression [37]. High and low iCBT program functionalities were also identified by therapists as facilitators and barriers, in building the working alliance in bCBT in the German and UK country sites of the E-COMPARED trial [36,70-72]. Although our findings remain preliminary and do not show a causal effect, further investigation concerning the effect of the digital program on the working alliance may be a fruitful direction for future research.

Collectively, our findings suggest that blending face-to-face CBT with an iCBT program may enhance the working alliance and treatment outcomes for depression. These findings hold important implications for clinical practice, especially following the COVID-19 pandemic that resulted in major shifts from in-person care to blended health care provision. The findings of this study suggest that a blended approach may enhance rather than worsen mental health care. Our study’s findings regarding the interaction between system usability and the working alliance in terms of treatment outcomes represent a preliminary step to quantitively understand the influence of the digital program and its role in how the working alliance is experienced. While further research is required to explore digital taxonomies that contribute toward fostering the working alliance in bCBT, our findings build on previous qualitative research [29,34,36,68] to explore a conceptualization of the working alliance that goes beyond the client and the therapist in order to consider the role of the digital program. The impact of the digital program on the working alliance may support the case of employing digital navigators who can help clients to use the intervention and troubleshoot technology and program usability issues, and remove the added burden of managing program-related problems that would otherwise fall on the therapist [70,72,73].

We propose 4 directions for future research. First, future research is required to build a comprehensive understanding of what, how, and when digital features (eg, usage, interface, interactivity, and accessibility) influence the working alliance [36]. Second, psychometric scales measuring the working alliance in bCBT should be adapted or developed to conceptually reflect a construct that also incorporates the client-program working alliance [42]. Third, the working alliance should be investigated early in the intervention and across multiple stages of treatment [74]. Fourth, future research should investigate if our results can be replicated across different DMHIs and treatment dosages.

### Limitations

Several study limitations should be noted. First, working alliance data were collected at a single point that corresponded with 3-month assessments. While this is common in clinical trials [25,58], the measurement of the alliance is recommended early in treatment within the first 5 sessions and at different points across treatment [74-77]. However, the number of face-to-face sessions varied between the 9 country sites (eg, 5 to 10 sessions), which would have posed significant challenges for the systematic data collection required in a clinical trial [54]. Second, the study engaged in multiple comparisons, which may have increased the risk of type 1 error (a positive result may be due to chance). However, given the exploratory nature of this analysis and the fact that different outcomes are likely to be highly correlated, a multiple adjustment comparison was not deemed necessary [78]. Third, the results of the analysis are valid under the MAR assumption, which we believe to be plausible because the effect of country sites appears to influence the missingness of the main outcome variables, stemming from country-specific data collection procedures and experiences. This is supported by chi-square analyses that indicate significantly higher rates of missing data for the PHQ-9 and WAI-SR-C across some countries compared to others. Nevertheless, it should be noted that this paper cannot rule out that data are missing not at random. Future research can explore this further using a sensitivity analysis. Fourth, the heterogeneity of interventions offered in the TAU group limits the study from conclusively tying causation to a specific comparator intervention. However, it should be noted that interventions offered by services in TAU were regarded as evidence-based, largely consisting of CBT and psychopharmacological interventions [35]. This may reduce the limitations associated with the multiple treatments offered in TAU [66,79] and adhering to the pragmatic trial’s ancillary objective to not impose specific constraints on clients and clinicians concerning data collection [79]. However, additional steps were also taken to address this limitation by conducting a subanalysis with a subset of trial country sites that only offered face-to-face CBT in TAU. The findings showed comparable results to those of the main analysis, highlighting that the addition of iCBT to face-to-face CBT may improve the quality of the working alliance. Fifth, another potential limitation is related to the variation in how bCBT was delivered across the trial’s country sites, concerning the number of sessions and the types of iCBT programs delivered, across different country sites. However, it should be noted that the study was focused on investigating the noninferiority of blending CBT given that there is a sufficient level of evidence concerning key treatment components, such as the CBT approach, and different delivery formats, including in-person and internet-based delivery of CBT for depression [80,81]. Although the number of treatment sessions varied between settings, to our knowledge, there is no evidence to suggest that the number of sessions of CBT effect the client-therapist alliance as the alliance is typically developed early in treatment and within the first 5 sessions [74-77]. Moreover, another study exploring the usage of different components of bCBT and treatment engagement when compared to intended use in the E-COMPARED study concluded that personalized blended care was more suitable compared to attempting to achieve a standardized optimal blend [69]. Variations in the number of treatment sessions described may enable a pragmatic understanding of the working alliance in bCBT interventions in real-world clinical settings [66].

### Conclusions

To our knowledge, this is the first study to show that bCBT may enhance the working alliance when compared to routine care for depression and when compared to face-to-face CBT. The working alliance in bCBT was also associated with clinical improvements in depression, which appear to be enhanced by good program usability. Collectively, our findings appear to add further weight to the view that the addition of iCBT to face-to-face CBT may positively augment experiences of the working alliance.

## Appendix

Multimedia Appendix 1 Supplementary methods and results that include information on: Participants' country of birth, information on missing data, medians and IQR for working alliance, participant characteristics, depression and system usability scores, trial profile, and results of the sensitivity analysis and subgroup analysis.

Multimedia Appendix 2 CONSORT-eHEALTH checklist (V.1.6.1).

## Abbreviations

bCBT: blended cognitive behavioral therapy
CBT: cognitive behavioral therapy
DMHI: digital mental health intervention
DSM-IV: Diagnostic and Statistical Manual of Mental Disorders IV
E-COMPARED: European Comparative Effectiveness Research on Blended Depression Treatment versus Treatment-as-usual
iCBT: internet-delivered cognitive behavioral therapy
ITT: intention to treat
MAR: missing at random
M.I.N.I: MINI International Neuropsychiatric Interview
PHQ-9: Patient Health Questionnaire-9
SUS-C: System Usability Scale-Client
TAU: treatment as usual
WAI-SR-C: Working Alliance Inventory-Short Revised–Client

## References

[1] GBD 2017 DiseaseInjury IncidencePrevalence Collaborators (2018). Global, regional, and national incidence, prevalence, and years lived with disability for 354 diseases and injuries for 195 countries and territories, 1990-2017: a systematic analysis for the Global Burden of Disease Study 2017. Lancet.

[2] Fact Sheet: Suicide. World Health Organization.

[3] Kohn R, Saxena S, Levav I, Saraceno B (2004). The treatment gap in mental health care. Bull World Health Organ.

[4] Fairburn CG, Patel V (2017). The impact of digital technology on psychological treatments and their dissemination. Behav Res Ther.

[5] Torous J, Jän Myrick K, Rauseo-Ricupero N, Firth J (2020). Digital Mental Health and COVID-19: Using Technology Today to Accelerate the Curve on Access and Quality Tomorrow. JMIR Ment Health.

[6] Kaltenthaler E, Parry G, Beverley C (2004). Computerized Cognitive Behaviour Therapy: A Systematic Review. Behav. Cogn. Psychother.

[7] Ruwaard J, Lange A, Schrieken B, Emmelkamp P (2011). Efficacy and effectiveness of online cognitive behavioral treatment: a decade of interapy research. Stud Health Technol Inform.

[8] Foroushani P, Schneider J, Assareh N (2011). Meta-review of the effectiveness of computerised CBT in treating depression. BMC Psychiatry.

[9] Ivarsson D, Blom M, Hesser H, Carlbring P, Enderby P, Nordberg R, Andersson G (2014). Guided internet-delivered cognitive behavior therapy for post-traumatic stress disorder: A randomized controlled trial. Internet Interventions.

[10] Cuijpers P, Donker T, Johansson R, Mohr DC, van Straten A, Andersson G (2011). Self-guided psychological treatment for depressive symptoms: a meta-analysis. PLoS One.

[11] Karyotaki E, Ebert DD, Donkin L, Riper H, Twisk J, Burger S, Rozental A, Lange A, Williams AD, Zarski AC, Geraedts A, van Straten A, Kleiboer A, Meyer B, Ünlü Ince B, Buntrock C, Lehr D, Snoek FJ, Andrews G, Andersson G, Choi I, Ruwaard J, Klein JP, Newby JM, Schröder J, Laferton JA, Van Bastelaar K, Imamura K, Vernmark K, Boß L, Sheeber LB, Kivi M, Berking M, Titov N, Carlbring P, Johansson R, Kenter R, Perini S, Moritz S, Nobis S, Berger T, Kaldo V, Forsell Y, Lindefors N, Kraepelien M, Björkelund C, Kawakami N, Cuijpers P (2018). Do guided internet-based interventions result in clinically relevant changes for patients with depression? An individual participant data meta-analysis. Clin Psychol Rev.

[12] Andrews G, Basu A, Cuijpers P, Craske M, McEvoy P, English C, Newby J (2018). Computer therapy for the anxiety and depression disorders is effective, acceptable and practical health care: An updated meta-analysis. J Anxiety Disord.

[13] Josephine K, Josefine L, Philipp D, David E, Harald B (2017). Internet- and mobile-based depression interventions for people with diagnosed depression: A systematic review and meta-analysis. J Affect Disord.

[14] Erbe D, Eichert H, Riper H, Ebert D (2017). Blending Face-to-Face and Internet-Based Interventions for the Treatment of Mental Disorders in Adults: Systematic Review. J Med Internet Res.

[15] Bordin ES (1979). The generalizability of the psychoanalytic concept of the working alliance. Psychotherapy: Theory, Research & Practice.

[16] Bordin ES, Horvath AO, Greenberg LS (1994). Theory and research on the therapeutic working alliance: New directions. The working alliance: Theory, research, and practice.

[17] Raue P, Goldfried M, Horvath AO, Greenberg LS (1994). The therapeutic alliance in cognitive-behavior therapy. The working alliance: Theory, research, and practice.

[18] Lambert MJ, Norcross JC, Goldfried MR (1992). Psychotherapy outcome research: Implications for integrative and eclectical therapists. Handbook of psychotherapy integration.

[19] Norcross JC, Lambert MJ (2011). Psychotherapy relationships that work II. Psychotherapy (Chic).

[20] Cameron S, Rodgers J, Dagnan D (2018). The relationship between the therapeutic alliance and clinical outcomes in cognitive behaviour therapy for adults with depression: A meta-analytic review. Clin Psychol Psychother.

[21] Pihlaja S, Stenberg J, Joutsenniemi K, Mehik H, Ritola V, Joffe G (2018). Therapeutic alliance in guided internet therapy programs for depression and anxiety disorders - A systematic review. Internet Interv.

[22] Gómez Penedo J, Berger T, Grosse Holtforth M, Krieger T, Schröder J, Hohagen F, Meyer B, Moritz S, Klein JP (2020). The Working Alliance Inventory for guided Internet interventions (WAI-I). J Clin Psychol.

[23] Heim E, Rötger A, Lorenz N, Maercker A (2018). Working alliance with an avatar: How far can we go with internet interventions?. Internet Interv.

[24] Henson P, Wisniewski H, Hollis C, Keshavan M, Torous J (2019). Digital mental health apps and the therapeutic alliance: initial review. BJPsych Open.

[25] Sucala M, Schnur JB, Constantino MJ, Miller SJ, Brackman EH, Montgomery GH (2012). The therapeutic relationship in e-therapy for mental health: a systematic review. J Med Internet Res.

[26] Berger T (2017). The therapeutic alliance in internet interventions: A narrative review and suggestions for future research. Psychother Res.

[27] Wehmann E, Köhnen M, Härter M, Liebherz S (2020). Therapeutic Alliance in Technology-Based Interventions for the Treatment of Depression: Systematic Review. J Med Internet Res.

[28] Hayati R, Bastani P, Kabir M, Kavosi Z, Sobhani G (2018). Scoping literature review on the basic health benefit package and its determinant criteria. Global Health.

[29] Tremain H, McEnery C, Fletcher K, Murray G (2020). The Therapeutic Alliance in Digital Mental Health Interventions for Serious Mental Illnesses: Narrative Review. JMIR Ment Health.

[30] Vernmark K, Hesser H, Topooco N, Berger T, Riper H, Luuk L, Backlund L, Carlbring P, Andersson G (2019). Working alliance as a predictor of change in depression during blended cognitive behaviour therapy. Cogn Behav Ther.

[31] Kooistra L, Ruwaard J, Wiersma J, van Oppen P, Riper H (2020). Working Alliance in Blended Versus Face-to-Face Cognitive Behavioral Treatment for Patients with Depression in Specialized Mental Health Care. J Clin Med.

[32] Askjer S, Mathiasen K (2021). The working alliance in blended versus face-to-face cognitive therapy for depression: A secondary analysis of a randomized controlled trial. Internet Interv.

[33] Barazzone N, Cavanagh K, Richards D (2012). Computerized cognitive behavioural therapy and the therapeutic alliance: a qualitative enquiry. Br J Clin Psychol.

[34] Clarke J, Proudfoot J, Whitton A, Birch M, Boyd M, Parker G, Manicavasagar V, Hadzi-Pavlovic D, Fogarty A (2016). Therapeutic Alliance With a Fully Automated Mobile Phone and Web-Based Intervention: Secondary Analysis of a Randomized Controlled Trial. JMIR Ment Health.

[35] Kleiboer A, Smit J, Bosmans J, Ruwaard J, Andersson G, Topooco N, Berger T, Krieger T, Botella C, Baños R, Chevreul K, Araya R, Cerga-Pashoja A, Cieślak R, Rogala A, Vis C, Draisma S, van Schaik A, Kemmeren L, Ebert D, Berking M, Funk B, Cuijpers P, Riper H (2016). European COMPARative Effectiveness research on blended Depression treatment versus treatment-as-usual (E-COMPARED): study protocol for a randomized controlled, non-inferiority trial in eight European countries. Trials.

[36] Doukani A, Free C, Michelson D, Araya R, Montero-Marin J, Smith S, Cerga-Pashoja A, Kakuma R (2020). Towards a conceptual framework of the working alliance in a blended low-intensity cognitive behavioural therapy intervention for depression in primary mental health care: a qualitative study. BMJ Open.

[37] Barceló-Soler A, García-Campayo J, Araya R, Doukani A, Gili M, García-Palacios A, Mayoral F, Montero-Marin J (2023). Working alliance in low-intensity internet-based cognitive behavioral therapy for depression in primary care in Spain: A qualitative study. Front Psychol.

[38] Mathiasen K, Andersen TE, Riper H, Kleiboer AAM, Roessler KK (2016). Blended CBT versus face-to-face CBT: a randomised non-inferiority trial. BMC Psychiatry.

[39] Kroenke K, Spitzer RL, Williams JBW (2001). The PHQ-9: validity of a brief depression severity measure. J Gen Intern Med.

[40] Lecrubier Y, Sheehan D, Weiller E, Amorim P, Bonora I, Sheehan KH, Janavs J, Dunbar G (2020). The Mini International Neuropsychiatric Interview (MINI). A short diagnostic structured interview: reliability and validity according to the CIDI. Eur. psychiatr.

[41] van der Vaart R, Witting M, Riper H, Kooistra L, Bohlmeijer E, van Gemert-Pijnen L (2014). Blending online therapy into regular face-to-face therapy for depression: content, ratio and preconditions according to patients and therapists using a Delphi study. BMC Psychiatry.

[42] Herrero R, Vara M, Miragall M, Botella C, García-Palacios Azucena, Riper H, Kleiboer Annet, Baños Rosa Mª (2020). Working Alliance Inventory for Online Interventions-Short Form (WAI-TECH-SF): The Role of the Therapeutic Alliance between Patient and Online Program in Therapeutic Outcomes. Int J Environ Res Public Health.

[43] Sheehan D, Lecrubier Y, Harnett Sheehan K, Janavs J, Weiller E, Keskiner A, Schinka J, Knapp E, Sheehan M, Dunbar G (1997). The validity of the Mini International Neuropsychiatric Interview (MINI) according to the SCID-P and its reliability. European Psychiatry.

[44] Rohde P, Lewinsohn PM, Seeley JR (1997). Comparability of telephone and face-to-face interviews in assessing axis I and II disorders. Am J Psychiatry.

[45] Ruskin PE, Reed S, Kumar R, Kling MA, Siegel E, Rosen M, Hauser P (1998). Reliability and acceptability of psychiatric diagnosis via telecommunication and audiovisual technology. Psychiatr Serv.

[46] Horvath AO, Greenberg LS (1989). Development and validation of the Working Alliance Inventory. Journal of Counseling Psychology.

[47] Cahill J, Barkham M, Hardy G, Gilbody S, Richards D, Bower P, Audin K, Connell J (2008). A review and critical appraisal of measures of therapist-patient interactions in mental health settings. Health Technol Assess.

[48] Fenton L, Cecero J, Nich C, Frankforter T, Carroll K (2001). Perspective is everything: the predictive validity of six working alliance instruments. J Psychother Pract Res.

[49] Luborsky L, Barber J, Siqueland L, Johnson S, Najavits L, Frank A, Daley D (1996). The Revised Helping Alliance Questionnaire (HAq-II) : Psychometric Properties. J Psychother Pract Res.

[50] Wittkampf KA, Naeije L, Schene AH, Huyser J, van Weert HC (2007). Diagnostic accuracy of the mood module of the Patient Health Questionnaire: a systematic review. Gen Hosp Psychiatry.

[51] Brooke J, Jordan PW, Thomas B, McClelland IL, Weerdmeester B (1996). SUS: A 'Quick and Dirty' Usability Scale. Usability Evaluation In Industry.

[52] Bangor A, Kortum PT, Miller JT (2008). An Empirical Evaluation of the System Usability Scale. International Journal of Human-Computer Interaction.

[53] Mol M, van Schaik A, Dozeman E, Ruwaard J, Vis C, Ebert D, Etzelmueller A, Mathiasen K, Moles B, Mora T, Pedersen C, Skjøth M, Pensado L, Piera-Jimenez J, Gokcay D, Ince B, Russi A, Sacco Y, Zanalda E, Zabala A, Riper H, Smit J (2020). Dimensionality of the system usability scale among professionals using internet-based interventions for depression: a confirmatory factor analysis. BMC Psychiatry.

[54] Ranganathan P, Pramesh C, Aggarwal R (2016). Common pitfalls in statistical analysis: Intention-to-treat versus per-protocol analysis. Perspect Clin Res.

[55] IBM SPSS Statistics 26. IBM Corp.

[56] Stata Statistical Software: Release 16. Stata Corp.

[57] Hayes AF (2017). Introduction to Mediation, Moderation, and Conditional Process Analysis (Second Edition): A Regression-Based Approach.

[58] Kirkwood BR, Stern JAC (2003). Essential Medical Statistics, 2nd Edition.

[59] Carpenter JR, Kenward MG (2013). Multiple Imputation and its Application.

[60] von Hippel PT (2009). 8. How to Impute Interactions, Squares, and other Transformed Variables. Sociological Methodology.

[61] de Souza R, Eisen R, Perera S, Bantoto B, Bawor M, Dennis B, Samaan Z, Thabane L (2016). Best (but oft-forgotten) practices: sensitivity analyses in randomized controlled trials. Am J Clin Nutr.

[62] Thabane L, Mbuagbaw L, Zhang S, Samaan Z, Marcucci M, Ye C, Thabane M, Giangregorio L, Dennis B, Kosa D, Borg Debono V, Dillenburg R, Fruci V, Bawor M, Lee J, Wells G, Goldsmith C (2013). A tutorial on sensitivity analyses in clinical trials: the what, why, when and how. BMC Med Res Methodol.

[63] Parpia S, Morris T, Phillips M, Wykoff C, Steel D, Thabane L, Bhandari M, Chaudhary V, Retina Evidence Trials InterNational Alliance (R.E.T.I.N.A.) Study Group (2022). Sensitivity analysis in clinical trials: three criteria for a valid sensitivity analysis. Eye (Lond).

[64] Morris TP, Kahan BC, White IR (2014). Choosing sensitivity analyses for randomised trials: principles. BMC Med Res Methodol.

[65] Burke J, Sussman J, Kent D, Hayward R (2015). Three simple rules to ensure reasonably credible subgroup analyses. BMJ.

[66] Dawson L, Zarin D, Emanuel E, Friedman L, Chaudhari B, Goodman S (2009). Considering usual medical care in clinical trial design. PLoS Med.

[67] Farrokhyar F, Skorzewski P, Phillips M, Garg S, Sarraf D, Thabane L, Bhandari M, Chaudhary V, Retina Evidence Trials InterNational Alliance (R.E.T.I.N.A.) Study Group (2022). When to believe a subgroup analysis: revisiting the 11 criteria. Eye (Lond).

[68] Doukani A, Free C, Araya R, Michelson D, Cerga-Pashoja A, Kakuma BR (2022). Practitioners' experience of the working alliance in a blended cognitive-behavioural therapy intervention for depression: qualitative study of barriers and facilitators. BJPsych Open.

[69] Kemmeren LL, van Schaik A, Smit JH, Ruwaard J, Rocha A, Henriques M, Ebert DD, Titzler I, Hazo J, Dorsey M, Zukowska K, Riper H (2019). Unraveling the Black Box: Exploring Usage Patterns of a Blended Treatment for Depression in a Multicenter Study. JMIR Ment Health.

[70] Titzler I, Saruhanjan K, Berking M, Riper H, Ebert D (2018). Barriers and facilitators for the implementation of blended psychotherapy for depression: A qualitative pilot study of therapists' perspective. Internet Interv.

[71] Titzler I, Berking M, Schlicker S, Riper H, Ebert D (2020). Barriers and Facilitators for Referrals of Primary Care Patients to Blended Internet-Based Psychotherapy for Depression: Mixed Methods Study of General Practitioners' Views. JMIR Ment Health.

[72] Cerga-Pashoja A, Doukani A, Gega L, Walke J, Araya R (2020). Added value or added burden? A qualitative investigation of blending internet self-help with face-to-face cognitive behaviour therapy for depression. Psychother Res.

[73] Wisniewski H, Torous J (2020). Digital navigators to implement smartphone and digital tools in care. Acta Psychiatr Scand.

[74] Crits-Christoph P, Gibbons MBC, Hamilton J, Ring-Kurtz S, Gallop R (2011). The dependability of alliance assessments: the alliance-outcome correlation is larger than you might think. J Consult Clin Psychol.

[75] Piper W, Azim H, Joyce A, McCallum M (1991). Transference interpretations, therapeutic alliance, and outcome in short-term individual psychotherapy. Arch Gen Psychiatry.

[76] Eames V, Roth A (2000). Patient attachment orientation and the early working alliance-a study of patient and therapist reports of alliance quality and ruptures. Psychother Res.

[77] Norcross JC, Wampold BE (2011). Evidence-based therapy relationships: research conclusions and clinical practices. Psychotherapy (Chic).

[78] Rothman K (1990). No Adjustments Are Needed for Multiple Comparisons. Epidemiology.

[79] Giraudeau B, Caille A, Eldridge S, Weijer C, Zwarenstein M, Taljaard M (2022). Heterogeneity in pragmatic randomised trials: sources and management. BMC Med.

[80] Karyotaki E, Efthimiou O, Miguel C, Bermpohl F, Furukawa T, Cuijpers P, Riper H, Patel V, Mira A, Gemmil A, Yeung A, Lange A, Williams A, Mackinnon A, Geraedts A, van Straten A, Meyer B, Björkelund C, Knaevelsrud C, Beevers C, Botella C, Strunk D, Mohr D, Ebert D, Kessler D, Richards D, Littlewood E, Forsell E, Feng F, Wang F, Andersson G, Hadjistavropoulos H, Christensen H, Ezawa I, Choi I, Rosso I, Klein J, Shumake J, Garcia-Campayo J, Milgrom J, Smith J, Montero-Marin J, Newby J, Bretón-López J, Schneider J, Vernmark K, Bücker L, Sheeber L, Warmerdam L, Farrer L, Heinrich M, Huibers M, Kivi M, Kraepelien M, Forand N, Pugh N, Lindefors N, Lintvedt O, Zagorscak P, Carlbring P, Phillips R, Johansson R, Kessler R, Brabyn S, Perini S, Rauch S, Gilbody S, Moritz S, Berger T, Pop V, Kaldo V, Spek V, Forsell Y, Individual Patient Data Meta-Analyses for Depression (IPDMA-DE) Collaboration (2021). Internet-Based Cognitive Behavioral Therapy for Depression: A Systematic Review and Individual Patient Data Network Meta-analysis. JAMA Psychiatry.

[81] Kambeitz-Ilankovic L, Rzayeva U, Völkel L, Wenzel J, Weiske J, Jessen F, Reininghaus U, Uhlhaas P, Alvarez-Jimenez M, Kambeitz J (2022). A systematic review of digital and face-to-face cognitive behavioral therapy for depression. NPJ Digit Med.

