# Supplementary material for: Comparison of the Working Alliance in Blended Cognitive Behavioral Therapy and Treatment as Usual for Depression in Europe: Secondary Data Analysis of the E-COMPARED Randomized Controlled Trial
Source: J Med Internet Res. 2024 May 31;26:e47515. doi: 10.2196/47515 (PMC11179025; doi:10.2196/47515)
Supplement: Multimedia Appendix 1 [file jmir_v26i1e47515_app1.docx]

**Multimedia Appendix 1.** Supplementary data and results.

**Methods S1.** Information on missing data.

| **Table S1.** Missing and complete data for WAI-SR-C and PHQ-9 scores at 3 months assessment across treatment conditions | | | | |
| --- | --- | --- | --- | --- |
|  | **WAI-SR-C (N=943)** | | **PHQ-9 (N=943)** | |
|  | *Missing* | *Complete* | *Missing* | *Complete* |
| **b-CBT** | 141 | 335 | 107 | 369 |
| **TAU** | 204 | 263 | 88 | 379 |
| Abbreviations: b-CBT, blended cognitive behavioural therapy; PHQ-9, Patient health questionnaire-9; TAU, treatment as usual, WAI-SR-C, working alliance inventory-short revised-client. | | | | |

| **Table S2.** Tabulation of missing and complete data for PHQ-9 data at 3-month assessments across country-sites on the E-COMPARED trial | | | |
| --- | --- | --- | --- |
| **Country-site** | **Missing** | **Complete** | **Total** |
| Germany | 9 | 164 | 173 |
| Sweden | 16 | 125 | 141 |
| Netherlands | 19 | 83 | 102 |
| UK | 25 | 76 | 101 |
| Spain | 35 | 92 | 127 |
| France | 26 | 79 | 105 |
| Switzerland | 6 | 44 | 50 |
| Poland | 38 | 46 | 84 |
| Denmark | 21 | 39 | 60 |
| Total | 195 | 748 | 943 |
| Abbreviation: PHQ, Patient health questionnaire-9 | | | |

| **Table S3.** Tabulation of missing and complete data for WAI-SR-C at 3-month follow-ups across country-sites on the E-COMPARED trial | | | |
| --- | --- | --- | --- |
| **Country-site** | **Missing** | **Complete** | **Total** |
| Germany | 43 | 130 | 173 |
| Sweden | 85 | 56 | 141 |
| Netherlands | 23 | 79 | 102 |
| UK | 47 | 54 | 101 |
| Spain | 39 | 88 | 127 |
| France | 34 | 71 | 105 |
| Switzerland | 6 | 44 | 50 |
| Poland | 43 | 41 | 84 |
| Denmark | 25 | 35 | 60 |
| Total | 345 | 598 | 943 |
| Abbreviation: WAI-SR-C, working alliance inventory-short revised-client | | | |

| **Table S4.** Comparison of missingness of data (missing vs complete cases) for WAI-SR-C across country-sites, derived from different models | |
| --- | --- |
| **Models** | **X² (df, N=) X² value, P level** |
| Germany vs Netherlands | X²(1, N=943)=9.71, P = .002 |
| Germany vs UK | X²(1, N=943)=4.83, P = .028 |
| Germany vs Spain | X²(1, N=943)=2.19, P = .139 |
| Germany vs France | X²(1, N=943)=0.90, P = .343 |
| Germany vs Switzerland | X²(1, N=943)=13.76, P = .000 |
| Germany vs Poland | X²(1, N=943)=8.49, P = .004 |
| Germany vs Denmark | X²(1, N=943)=0.71, P = 398 |
| Abbreviation: WAI-SR-C, working alliance inventory-short revised-client | |

| **Table S5.** Comparison of missingness of data (missing vs complete cases) for PHQ-9 across country-sites, derived from different models | |
| --- | --- |
| **Models** | **X² (df, N=) X² value, P level** |
| Germany vs Sweden | X²(1, N=943)=8.80, P = .003 |
| Germany vs Netherlands | X²(1, N=943)=0.29, P = .588 |
| Germany vs UK | X²(1, N=943)=1.14, P = .285 |
| Germany vs Spain | X²(1, N=943)=4.24, P = .040 |
| Germany vs France | X²(1, N=943)=1.20, P = .273 |
| Germany vs Switzerland | X²(1, N=943)=2.43, P = .119 |
| Germany vs Poland | X²(1, N=943)=33.91, P = .000 |
| Germany vs Denmark | X²(1, N=943)=8.01, P = .005 |
| Abbreviation: PHQ, Patient health questionnaire-9 | |

## Results S1. Trial profile diagram, means, and standard deviation of working alliance and system usability scores.

**Figure S1.** E-COMPARED Trial Profile

3 - 6 - 12 month (n=344 - 319 - 285)

DE =84 – 77 - 79

SWE =62 – 60 - 60

NL =42 – 41 - 35

UK =36 – 36 - 20

ESP =53 – 41- 28

FR =39 – 30 - 30

SWI =22 – 14 - 9

PL =24 – 28 - 31

DK =20 – 16 – 14

3 - 6 - 12 month (n=345 – 293 - 274)

DE =83 – 81 - 76

SWE =63 – 50 - 53

NL =42 – 32 - 34

UK =43 – 37 - 16

ESP =53 – 46 - 32

FR =44 – 37 - 36

SWI =22 – 13 - 10

PL =23 – 19 - 24

DK =20 – 19 - 12

## Follow-Ups

Allocated to b-CBT (n=476)

DE =86

SWE =73

NL =53

UK =49

ESP =64

FR =51

SWI =26

PL =42

DK =32

Allocated to TAU (n=467)

DE =87

SWE =68

NL =49

UK =52

ESP =63

FR =54

SWI =24

PL =42

DK =28

Randomized (n=943)

## Enrollment

Assessed for eligibility (n=1755)

Excluded (n=802)

- Not meeting inclusion criteria (n=384)
- Declined to participate (n=244)
- Other reasons (n=174)

Baseline assessments completed (n=953)

Excluded (n=10)

- Withdrew (n=10)

## Allocation

**Abbreviations**: b-CBT, blended-Cognitive Behavioural Therapy; DE, Germany; DK, Denmark; ESP, Spain; FR, France; NL, Netherlands; ESP, Spain; NL, Netherlands; PL, Poland; SWE, Sweden; SWI, Switzerland; TAU, Treatment as Usual; UK, United Kingdom;

| Table S6. Median (IQR) of WAI-SR-C composite and sub-scale scores (goals, task, bond), and SUS-C total scores | | | |
| --- | --- | --- | --- |
|  | **b-CBT** | **Control** | **Total** |
| **WAI-SR-C** |  |  |  |
| **Composite** | 47.42 (6) | 42 (8) | 46 (9.2) |
| **Goals** | 16.08 (3.4) | 14 (3.9) | 15.50 (4.7) |
| **Task** | 14.45 (3) | 12.83 (4) | 14 (4) |
| **Bond** | 17 (4) | 15.43 (3) | 16 (3.7) |
| **SUS-C total^d^** | 42 (9) | n/a | n/a |
| Abbreviations: b-CBT, blended-cognitive behavioural therapy; IQR, interquartile range; SUS-C, system usability scale-client; WAI-SR-C, working alliance inventory-short revised-client.  ^d^SUS-C data was only collected in the b-CBT arm owing to the use of a digital program. | | | |

| Table S7. Median (IQR) of composite WAI-SR-C by each country-site | | | |
| --- | --- | --- | --- |
| **Country** | **b-CBT** | **Control** | **Total** |
| **Germany** | 48.45 (10.0) | 40.00 (10.2) | 44 (10.5) |
| **Sweden** | 47.17 (5.5) | n/a | 47.17 (5.5) |
| **Netherlands** | 47.05 (5.4) | 43.57 (7.9) | 46 (7.3) |
| **United Kingdom** | 47.00 (4.5) | 41.29 (8.2) | 45 (8.4) |
| **Spain** | 47.50 (7.8) | 41.46 (10) | 45 (12) |
| **France** | 47.00 (7.0) | 43.95 (8.5) | 46 (8.3) |
| **Switzerland** | 48.00 (7.3) | 47 (12.8) | 47.85 (9.0) |
| **Poland** | 47.98 (7.2) | 43.33 (12.2) | 47.17 (11.7) |
| **Denmark** | 45.91 (7.3) | 42.12 (5.8) | 44.36 (7.2) |
| Abbreviations: b-CBT, blended-cognitive behavioural therapy; IQR, interquartile range; SUS-C, system usability scale-client; WAI-SR-C, working alliance inventory-short revised-client; | | | |

## Table S8. Median (IQR) of system usability scale by each country-site

| **Country** | **b-CBT** |
| --- | --- |
| **Germany** | 45.50 (7) |
| **Sweden** | 42 (6) |
| **Netherlands** | 40 (10) |
| **United Kingdom** | 42.13 (5) |
| **Spain** | 41.00 (8) |
| **France** | 41.00 (9) |
| **Switzerland** | 44.71 (5) |
| **Poland** | 42.11 (5) |
| **Denmark** | 41.90 (3) |
| Abbreviations: b-CBT, blended-cognitive behavioral therapy; IQR, interquartile range | |

## Methods S2. Participant information.

| Table S9. Self-reported country of birth across nine trial country-sites | |
| --- | --- |
| Country-site (n=943) | Country of Birth |
| Germany (n=173) | Germany, n=16, Bosnia and Herzegovina (n=1), Bulgaria (n=1), China, (n=1), Italy (n=1), Kazakhstan (n=1), Romania, Russia (n=2), Serbia (n=1), Indonesia (n=1), Ukraine (n=1), and USA (n=1) |
| Sweden (141) | Sweden, n=133, Missing data, n=8. |
| Netherlands (102) | Netherlands (n=83), (Chile (n=1), Colombia (n=1), Iraq (n=1), Iran (n=1), Macedonie (n=1), Mexico (n=1), Morocco (n=6), Suriname (n=3), Turkey (n=4). |
| United Kingdom (101) | UK (n=95), China (n=2), Colombia (n=1), Romania (n=1), Sri Lanka (n=1), and Ukraine (n=1) |
| Spain (127) | Spain, (n=127), Paraguay (n=1) and Argentina (n=1) |
| French (105) | French (n=86), Algeria (n=3), Belgium (n=1), Côte d'Ivoire (n=2)  Madagascar (n=1), Morocco (n=4), Portugal (n=1), Martinique (n=2), Mauritius, Serbia (n=1), Syria (n=1), and Tunisia (n=2) |
| Switzerland (50) | Switzerland (n=44), Albania (n=2), Austria (n=1), Kosovo (n=1), Turkey, (n=1), missing data (n=1) |
| **Poland (84)** | Poland, n=84 |
| **Denmark (60)** | Denmark, n=60 |
| Abbreviations: b-CBT, blended-cognitive behavioral therapy; IQR, interquartile range | |

## Results S2. Summary of differences between the main analysis, and the sensitivity and sub-analysis

In addition to the results resulted for the sensitivity and subgroup in the main text, this section will provide additional differences that were observed for models pertaining to sub-scale scores, across the sensitivity analysis that employed a complete case analysis, the sub-group that only include five country-sites that offered face-to-face CBT in the TAU arm.

Accordingly, notable differences were as follows: Higher bond-subscales scores were associated with lower PHQ-9 scores across both blended-cognitive behavioural therapy (b-CBT) and treatment as usual (TAU) for both the sensitivity analysis (b-CBT: -0.22 (-0.40 to -0.34); TAU: -0.17 (-0.33 to -0.02)) and the sub-group analysis (b-CBT: -0.32 [-0.55 to -0.09]; TAU: -0.24 [-0.46 to -0.02]). Moreover, the goals and task-sub scores were not significantly associated with patient health quesyionnaire-9 (PHQ-9) scores in the sub-group. There interaction between WAI-SR-C and SUS-C on the association between WAI-SR-C composite scores and PHQ-9 at 3 months was not significant for composite, and all subscale models.

## Results S3. Results of sensitivity analysis using complete case analysis

### Sensitivity Analysis: Impact of Multiple Imputation on the Results

A sensitivity analysis was conducted using a complete case analysis, to understand the impact of multiple imputation on the results. The full findings of the sensitivity analysis are summarized below.

### Treatment Assignment as a Predictor for WAI-SR-C scores

Treatment assignment significantly predicted WAI-SR-C composite, goals, task and bond scores. Being allocated to b-CBT was related to higher WAI-SR-C composite and all subscale scores at 3- month follow-up assessments, when compared to participants in TAU.

### Association between PHQ-9 scores and WAI-SR-C Scores at 3-month assessments

Across both treatment arms, WAI-SR-C composite, goal, task and bond subscales scores were significantly associated with post-treatment PHQ-9 scores, in which lower PHQ-9 scores were associated with higher WAI-SR composite, goals and task scores. WAI-SR bond scores were not significantly associated with PHQ-9 scores in any of the arms.

### Testing the interaction between WAI-SR-C and SUS-C on the relationship between WAI-SR-C and PHQ-9, as 3-months

The association between the WAI-SR-C task sub-scale and PHQ-9 scores was moderated by SUS scores (*b* = 0.34, CI 95 -0.06 to -0.01; *P* = .006). Figure S2 shows a trend for an inverse association between composite WAI-SR-C and PHQ-9 scores among those with higher SUS-C scores. No significant interactions were observed in the other WAI-SR-C sub-scales scores.

| **Table S10.** Adjusted linear regression models of treatment assignment as a predictor for WAI-SR-C composite, and subscales (goals, task, bond) scores | | |
| --- | --- | --- |
| WAI-SR (outcome) | *B*  (95% CI) | *P V*alue |
| **Model 1**  Composite | 5.54 (3.81 to7.27) | <.001 |
| **Model 2**  Goals | 2.25 (1.61 to 2.88) | <.001 |
| **Model 3**  Task | 2.05 (1.42 to2.68) | <.001 |
| **Model 4**  Bond | 1.35 (0.73 to 1.96) | <.001 |
| Abbreviations: 95% CI, 95% confidence intervals; *B*, unstandardized beta; PHQ-9, patient health questionnaire-9; WAI-SR-C, working alliance inventory short-revised. | | |

| Table S11. Adjusted linear regression analysis models of the association between working alliance post-treatment (3-month) PHQ-9 scores and WAI-SR Composite, and subscales (goals, task, bond), across participants in the b-CBT and TAU. | | | | | |
| --- | --- | --- | --- | --- | --- |
| WAI-SR  (outcome) | b-CBT | | TAU | |  |
|  | *B* (95% CI) | *P* value | *B* (95% CI) | *P* value |  |
| **Model 1**  Composite | -0.12  (-0.18 to -0.05) | <.001 | -0.91  (-0.15 to -0.04) | .002 |  |
| **Model 2**  Goals | -0.27  (-0.45 to -0.09) | .004 | -0.20  (-0.34 to -0.05) | .008 |  |
| **Model 3**  Task | -0.39  (-0.55 to -0.22) | <.001 | -0.29  (-0.44 to -0.13) | <.000 |  |
| **Model 4**  Bond | -0.22  (-0.40 to -0.34) | .020 | -0.17  (-0.33 to -0.02) | .029 |  |
| Abbreviations: 95% CI, 95% confidence intervals; *B*, unstandardized beta; PHQ-9, patient health questionnaire-9; WAI-SR-C, working alliance inventory short revised-C.  WAI-SR-C, working alliance inventory short form; PHQ-9, patient health questionnaire-9; b-CBT, blended-cognitive behavioral therapy; TAU, treatment as usual; *B* unstandardized beta; 95% CI, 95% confidence intervals. | | | | | |

#

##
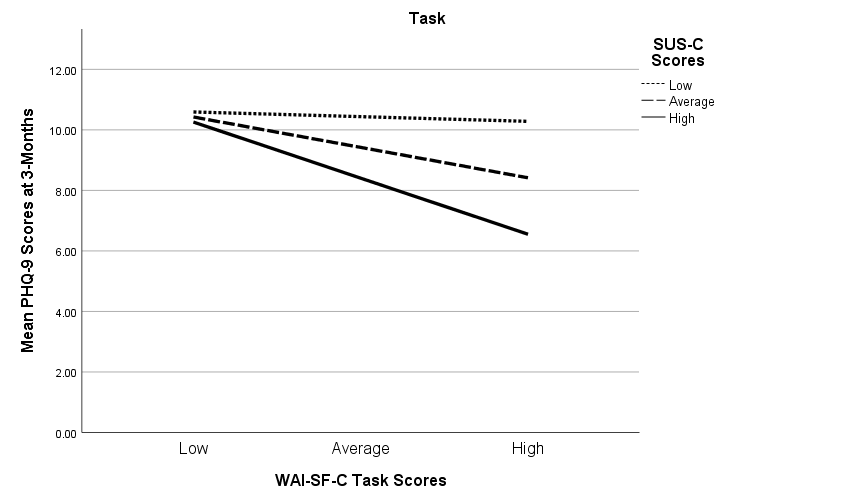


Abbreviation: PHQ-9, patient health questionnaire-9; SUS-C, system usability scale- client; WAI-SR-C, working alliance inventory-short revised-client.

**Figure S2.** Multiple line graph of the interaction between SUS-C on WAI-SR-C on the association between WAI-SR-C (composite and subscale scores, goals, task and bond) and PHQ-9 scores at 3 month assessment in the b-CBT arm

## Results S4. Results of sub-analysis using data from sites that only offered face-to-face

## Cognitive Behavioral Therapy (CBT) in the Treatment as Usual (TAU)

A sensitivity analysis was conducted using a complete case analysis, to understand the impact of treatment allocation in TAU on the results. The full findings of the sensitivity analysis are summarized below.

### Treatment Assignment as a Predictor for WAI-SR-C scores

Treatment assignment significantly predicted WAI-SR composite, goals, task and bond scores. Being allocated to b-CBT was related to higher WAI-SR-C composite and all subscale scores at 3- month follow-up assessments, when compared to participants in TAU.

### Association Between PHQ-9 Scores and WAI-SR-C Scores at 3 month-assessements

Across both treatment arms, WAI-SR-C composite, goals and task subscales scores were significantly associated with post-treatment PHQ-9 scores, in which lower PHQ-9 scores were associated with higher WAI-SR composite, goals and task scores. While WAI-SR-C bond scores were not significantly associated with PHQ-9 scores in any of the arms. It should be noted that there were missing correlations between PHQ-9 measured at 3-months and marital status leave single vs widow, in the b-CBT arm, which was entered as a covariate.

### Testing the interaction between WAI-SR-C and SUS-C on the relationship between WAI-SR-C and PHQ-9, at 3-months assessments

The association between WAI-SR-C composite scores and PHQ-9 scores does not seem to be moderated by SUS (*b* = -0.018, CI 95: -0.05; 0.01; *P* = 0.253). Moreover, no significant interactions were observed in the other WAI-SR sub-scales scores.

| Table S12. Baseline characteristics of participants and WAI-SR-C and SUS scores at 3 month assessments. Figures are numbers (percentages) of participants unless otherwise indicated. | | | |
| --- | --- | --- | --- |
| Characteristic at baseline | b-CBT  N= 200 | TAU  *N=200* | Total  N= 400 |
| Mean (SD) min-max, age in years | 38.27 (13.75) 18-74 | 37.89  (13.76)  18-70 | 38.07 (13.74) 18-74 |
| Female, n (%) | 135 (68) | 135 (68) | 270 (68) |
| Marital status |  |  |  |
| Single | 73 (37) | 75 (38) | 148 (37) |
| Divorced | 28 (14) | 20 (10) | 48 (12) |
| Widowed | 0 | 2 (1.0) | 2 (0.5) |
| Living together | 39 (20) | 41 (21) | 80 (20) |
| Married | 60 (30) | 62 (31) | 122 (31) |
| Level of education* |  |  |  |
| Secondary School, equivalent | 32 (16) | 34 (17) | 66 (17) |
| College, equivalent | 81 (41) | 65 (33) | 146 (37) |
| University degree or higher | 87 (44) | 101 (51) | 188 (47) |
| Median (IQR) and min-max  of baseline PHQ-9 scores^c^ | 15. 67 (4.63)  2-27 | 15.48 (4.52)  5-26 | 15.57 (4.56) 5-27) |
| WAI-SR-C |  |  |  |
| Composite | 48.83 (7.73)  12-60 | 44.22 (7.57)  12-60 | 45.52 (7.75)  12-60 |
| Goals | 15.83 (2.98)  4-20 | 14.49 (3.02)  4-20 | 15.16 (3.07)  4-20 |
| Task | 14.76 (2.87)  4-20 | 14.18 (2.98)  4-20 | 14.47 (2.94)  4-20 |
| Bond | 16.24 (3.03)  4-14.7 | 15.55 (2.82)  4-20 | 15.89 (2.95)  4-24.7 |
| SUS-C^d^ | 40.37 (6.84)  14-50 | n/a | 40.37 (6.84)  14-50 |
| Abbreviations: b-CBT, blended cognitive behaviour therapy; SD, standard deviation; PHQ-9= patient health questionnaire-9; TAU, treatment as usual; SUS-C, system usability scale-client; WAI-SR-C, working alliance inventory-short revised-client.  Note: SD= standard deviation, IQR= Inter-quarter-range, b-CBT=blended cognitive behaviour therapy, TAU=treatment as usual and PHQ-9- patient health questionnaire-9.  *Data collected was in respect to what would be considered low, middle, and high level of education. Data was missing for n=1/ 943 (0.2%) in the b-CBT group.  aData collected was in respect to what would be considered low, middle, and high level of education in each setting. Data was missing for n=1/ 943 (0.2%) in the b-CBT arm.  c PHQ-9 severity cut-off points are: 5-9 indicate mild depression, 1-14 for moderate depression 15-19 moderately severe depression, and ≥ 20 for severe depression.27 | | | |

| Table S13. Median (IQR) of WAI-SR-C composite and sub-scale scores (goals, task, bond), and SUS-C total scores. | | | |
| --- | --- | --- | --- |
|  | **b-CBT** | **Control** | **Total** |
| **WAI-SR-C** |  |  |  |
| **Composite** | 48.83 (7.73)  12-60 | 44.22 (7.57)  12-60 | 45.52 (7.75)  12-60 |
| **Goals** | 15.83 (2.98)  4-20 | 14.49 (3.02)  4-20 | 15.16 (3.07)  4-20 |
| **Task** | 14.76 (2.87)  4-20 | 14.18 (2.98)  4-20 | 14.47 (2.94)  4-20 |
| **Bond** | 16.24 (3.03)  4-14.7 | 15.55 (2.82)  4-20 | 15.89 (2.95)  4-24.7 |
| **SUS-C total^d^** | 40.37 (6.84)  14-50 | n/a | 40.37 (6.84)  14-50 |
| Abbreviations: b-CBT, blended-cognitive behavioural therapy; IQR, interquartile range; SUS-C, system usability scale-client, WAI-SR-C, working alliance inventory-short revised-client.  ^d^SUS-C, data was only collected in the b-CBT arm owing to the use of a digital program. | | | |

| **Table S14.** Adjusted linear regression models of treatment assignment as a predictor for WAI-SR-C composite, and subscales (goals, task, bond) scores | | |
| --- | --- | --- |
| WAI-SR (outcome) | *B*  (95% CI) | *P V*alue |
| **Model 1**  Composite | 2.63 (1.13 to 4.12) | .001 |
| **Model 2**  Goals | 1.36 (0.79 to 1.94) | <.001 |
| **Model 3**  Task | 0.63 (0.07 to 1.18) | .028 |
| **Model 4**  Bond | 0.64 (0.07 to 1.21) | .029 |
| Abbreviations: 95% CI, 95% confidence intervals; *B*, unstandardized beta; PHQ-9, patient health questionnaire-9; WAI-SR-C, working alliance inventory short-revised-client; | | |

| Table S15. Linear regression models of associations between post-treatment PHQ-9 and WAI-SR-C composite and subscale (goals, task, bond) scores across b-CBT and TAU | | | | |
| --- | --- | --- | --- | --- |
| WAI-SR  (outcome) | b-CBT | | TAU | |
|  | *B* (95% CI) | *P* value | *B* (95% CI) | *P* value |
| **Model 1**  Composite | -0.16  (-0.25 to -0.07) | .001 | -0.09  (-0.18 to -0.01) | .028 |
| **Model 2**  Goals | -0.33  (-0.57 to -0.10) | .006 | -0.20  (-0.41 to 0.02) | .068 |
| **Model 3**  Task | -0.45  (-0.69 to -0.21) | <.001 | -0.20  (-0.43 to 0.02) | .073 |
| **Model 4**  Bond | -0.32  (-0.55 to -0.09) | .006 | -0.24  (-0.46 to -0.02) | .033 |
| Abbreviations: 95% CI, 95% confidence intervals; b-CBT, blended-cognitive behavioral therapy; PHQ-9, patient health questionnaire-9; TAU, treatment as usual; *B,* unstandardized beta; WAI-SR-C, working alliance inventory short-revised-client. | | | | |
