# Supplementary material for: Comparison of the Working Alliance in Blended Cognitive Behavioral Therapy and Treatment as Usual for Depression in Europe: Secondary Data Analysis of the E-COMPARED Randomized Controlled Trial
Source: J Med Internet Res. 2024 May 31;26:e47515. doi: 10.2196/47515 (PMC11179025; doi:10.2196/47515)
Supplement: Multimedia Appendix 2 [file jmir_v26i1e47515_app2.pdf]

CONSORT-EHEALTH (V 1.6.1) - Submission/Publication Form

The CONSORT-EHEALTH checklist is intended for authors of randomized trials evaluating web-based and Internet-based applications/interventions, including mobile interventions, electronic games (incl multiplayer games), social media, certain telehealth applications, and other interactive and/or networked electronic applications. Some of the items (e.g. all subitems under item 5 - description of the intervention) may also be applicable for other study designs.

The goal of the CONSORT EHEALTH checklist and guideline is to be
a) a guide for reporting for authors of RCTs,
b) to form a basis for appraisal of an ehealth trial (in terms of validity)

CONSORT-EHEALTH items/subitems are MANDATORY reporting items for studies published in the Journal of Medical Internet Research and other journals / scientific societies endorsing the checklist.

Items numbered 1., 2., 3., 4a., 4b etc are original CONSORT or CONSORT-NPT (non-pharmacologic treatment) items.
Items with Roman numerals (i., ii, iii, iv etc.) are CONSORT-EHEALTH extensions/clarifications.

As the CONSORT-EHEALTH checklist is still considered in a formative stage, we would ask that you also RATE ON A SCALE OF 1-5 how important/useful you feel each item is FOR THE PURPOSE OF THE CHECKLIST and reporting guideline (optional).

Mandatory reporting items are marked with a red *.
In the textboxes, either copy & paste the relevant sections from your manuscript into this form - please include any quotes from your manuscript in QUOTATION MARKS,
or answer directly by providing additional information not in the manuscript, or elaborating on why the item was not relevant for this study.

YOUR ANSWERS WILL BE PUBLISHED AS A SUPPLEMENTARY FILE TO YOUR PUBLICATION IN JMIR AND ARE CONSIDERED PART OF YOUR PUBLICATION (IF ACCEPTED).
Please fill in these questions diligently. Information will not be copyedited, so please use proper spelling and grammar, use correct capitalization, and avoid abbreviations.

DO NOT FORGET TO SAVE AS PDF _AND_ CLICK THE SUBMIT BUTTON SO YOUR ANSWERS ARE IN OUR DATABASE !!!

Citation Suggestion (if you append the pdf as Appendix we suggest to cite this paper in the caption):
Eysenbach G, CONSORT-EHEALTH Group
CONSORT-EHEALTH: Improving and Standardizing Evaluation Reports of Web-based and Mobile Health Interventions
J Med Internet Res 2011;13(4):e126

URL: http://www.jmir.org/2011/4/e126/
doi: 10.2196/jmir.1923
PMID: 22209829

asmae.doukani@googlemail.com Switch accounts

Not shared

Resubmit to save

* Indicates required question

Your name *

First Last

Asmae Doukani

Primary Affiliation (short), City, Country *

University of Toronto, Toronto, Canada

London School of Hygiene and Tropical Medici

Your e-mail address *

abc@gmail.com

asmae.doukani@lshtm.ac.uk

Title of your manuscript *

Provide the (draft) title of your manuscript.

Comparison of the Working Alliance in Blended Cognitive Behavioral Therapy and Treatment as Usual for Depression in Europe: Secondary Data Analysis of the E-COMPARED Randomized Controlled Trial

Name of your App/Software/Intervention *

If there is a short and a long/alternate name, write the short name first and add the long name in brackets.

iCBT (internet-based CBT)

Evaluated Version (if any)

e.g. "V1", "Release 2017-03-01", "Version 2.0.27913"

Your answer

Language(s) *

What language is the intervention/app in? If multiple languages are available, separate by comma (e.g. "English, French")

English, Danish, Dutch, French, German, Polish

URL of your Intervention Website or App

e.g. a direct link to the mobile app on app in appstore (itunes, Google Play), or URL of the website. If the intervention is a DVD or hardware, you can also link to an Amazon page.

Your answer

URL of an image/screenshot (optional)

Your answer

Accessibility *

Can an enduser access the intervention presently?

- ( ) access is free and open
- ( ) access only for special usergroups, not open
- ( ) access is open to everyone, but requires payment/subscription/in-app purchases
- (●) app/intervention no longer accessible
- ( ) Other:

Primary Medical Indication/Disease/Condition *

e.g. "Stress", "Diabetes", or define the target group in brackets after the condition, e.g. "Autism (Parents of children with)", "Alzheimers (Informal Caregivers of)"

Depression

Primary Outcomes measured in trial *

comma-separated list of primary outcomes reported in the trial

Depression

Secondary/other outcomes

Are there any other outcomes the intervention is expected to affect?

Your answer

Recommended "Dose" *

What do the instructions for users say on how often the app should be used?

- ○ Approximately Daily
- ◉ Approximately Weekly
- ○ Approximately Monthly
- ○ Approximately Yearly
- ○ "as needed"
- ○ Other:

Approx. Percentage of Users (starters) still using the app as recommended after 3 months *

- ○ unknown / not evaluated
- ○ 0-10%
- ○ 11-20%
- ○ 21-30%
- ○ 31-40%
- ○ 41-50%
- ○ 51-60%
- ○ 61-70%
- ○ 71%-80%
- ○ 81-90%
- ○ 91-100%
- ◉ Other: Not applicable. Most interventions ended after 3-months and particip[illegible]

Overall, was the app/intervention effective? *

- [ ] yes: all primary outcomes were significantly better in intervention group vs control
- [ ] partly: SOME primary outcomes were significantly better in intervention group vs control
- [ ] no statistically significant difference between control and intervention
- [ ] potentially harmful: control was significantly better than intervention in one or more outcomes
- [ ] inconclusive: more research is needed
- [x] Other: The intervention (blended-CBT) appears to show significantly higher v

Article Preparation Status/Stage *

At which stage in your article preparation are you currently (at the time you fill in this form)

- [ ] not submitted yet - in early draft status
- [ ] not submitted yet - in late draft status, just before submission
- [ ] submitted to a journal but not reviewed yet
- [ ] submitted to a journal and after receiving initial reviewer comments
- [x] submitted to a journal and accepted, but not published yet
- [ ] published
- [ ] Other:

Journal *

If you already know where you will submit this paper (or if it is already submitted), please provide the journal name (if it is not JMIR, provide the journal name under "other")

- ( ) not submitted yet / unclear where I will submit this
- (•) Journal of Medical Internet Research (JMIR)
- ( ) JMIR mHealth and UHealth
- ( ) JMIR Serious Games
- ( ) JMIR Mental Health
- ( ) JMIR Public Health
- ( ) JMIR Formative Research
- ( ) Other JMIR sister journal
- ( ) Other:

Is this a full powered effectiveness trial or a pilot/feasibility trial? *

- ( ) Pilot/feasibility
- (•) Fully powered

Manuscript tracking number *

If this is a JMIR submission, please provide the manuscript tracking number under "other" (The ms tracking number can be found in the submission acknowledgement email, or when you login as author in JMIR. If the paper is already published in JMIR, then the ms tracking number is the four-digit number at the end of the DOI, to be found at the bottom of each published article in JMIR)

- ( ) no ms number (yet) / not (yet) submitted to / published in JMIR
- (•) Other: 47515

TITLE AND ABSTRACT

1a) TITLE: Identification as a randomized trial in the title

1a) Does your paper address CONSORT item 1a? *

I.e does the title contain the phrase "Randomized Controlled Trial"? (if not, explain the reason under "other")

- (selected) yes
- Other:

1a-i) Identify the mode of delivery in the title

Identify the mode of delivery. Preferably use “web-based” and/or “mobile” and/or “electronic game” in the title. Avoid ambiguous terms like “online”, “virtual”, “interactive”. Use “Internet-based” only if Intervention includes non-web-based Internet components (e.g. email), use “computer-based” or “electronic” only if offline products are used. Use “virtual” only in the context of “virtual reality” (3-D worlds). Use “online” only in the context of “online support groups”. Complement or substitute product names with broader terms for the class of products (such as “mobile” or “smart phone” instead of “iphone”), especially if the application runs on different platforms.

	1	2	3	4	5	
subitem not at all important	○	○	●	○	○	essential

Clear selection

Does your paper address subitem 1a-i? *

Copy and paste relevant sections from manuscript title (include quotes in quotation marks "like this" to indicate direct quotes from your manuscript), or elaborate on this item by providing additional information not in the ms, or briefly explain why the item is not applicable/relevant for your study

“Blended Cognitive Behavioral Therapy" - Blended therapy is commonly used to refer to interventions that include a digital intervention with higher levels of professional support.

1a-ii) Non-web-based components or important co-interventions in title

Mention non-web-based components or important co-interventions in title, if any (e.g., “with telephone support”).

	1	2	3	4	5	
subitem not at all important	○	○	●	○	○	essential

Clear selection

Does your paper address subitem 1a-ii?

Copy and paste relevant sections from manuscript title (include quotes in quotation marks "like this" to indicate direct quotes from your manuscript), or elaborate on this item by providing additional information not in the ms, or briefly explain why the item is not applicable/relevant for your study

See 1a-11 for response regarding 'blended' CBT.

1a-iii) Primary condition or target group in the title

Mention primary condition or target group in the title, if any (e.g., “for children with Type I Diabetes”) Example: A Web-based and Mobile Intervention with Telephone Support for Children with Type I Diabetes: Randomized Controlled Trial

	1	2	3	4	5	
subitem not at all important	○	○	○	◉	○	essential

Clear selection

Does your paper address subitem 1a-iii? *

Copy and paste relevant sections from manuscript title (include quotes in quotation marks "like this" to indicate direct quotes from your manuscript), or elaborate on this item by providing additional information not in the ms, or briefly explain why the item is not applicable/relevant for your study

The focus of the study are adults with depression. "Blended Cognitive Behavioral Therapy and Treatment as Usual for Depression" – this section of the title indicates the target group.

1b) ABSTRACT: Structured summary of trial design, methods, results, and conclusions

NPT extension: Description of experimental treatment, comparator, care providers, centers, and blinding status.

1b-i) Key features/functionalities/components of the intervention and comparator in the METHODS section of the ABSTRACT

Mention key features/functionalities/components of the intervention and comparator in the abstract. If possible, also mention theories and principles used for designing the site. Keep in mind the needs of systematic reviewers and indexers by including important synonyms. (Note: Only report in the abstract what the main paper is reporting. If this information is missing from the main body of text, consider adding it)

	1	2	3	4	5	
subitem not at all important	○	○	○	○	◉	essential

Clear selection

Does your paper address subitem 1b-i? *

Copy and paste relevant sections from the manuscript abstract (include quotes in quotation marks "like this" to indicate direct quotes from your manuscript), or elaborate on this item by providing additional information not in the ms, or briefly explain why the item is not applicable/relevant for your study

"We conducted a secondary data analysis of the noninferiority E-COMPARED (European Comparative Effectiveness Research on Blended Depression Treatment versus Treatment-as-usual) trial comparing bCBT with TAU across 9 European countries." and "bCBT consisted of 6-20 sessions of bCBT (involving face-to-face sessions with a therapist and an internet-based program). TAU consisted of usual care for depression".

1b-ii) Level of human involvement in the METHODS section of the ABSTRACT

Clarify the level of human involvement in the abstract, e.g., use phrases like “fully automated” vs. “therapist/nurse/care provider/physician-assisted” (mention number and expertise of providers involved, if any). (Note: Only report in the abstract what the main paper is reporting. If this information is missing from the main body of text, consider adding it)

	1	2	3	4	5	
subitem not at all important	○	○	○	◉	○	essential

Clear selection

Does your paper address subitem 1b-ii?

Copy and paste relevant sections from the manuscript abstract (include quotes in quotation marks "like this" to indicate direct quotes from your manuscript), or elaborate on this item by providing additional information not in the ms, or briefly explain why the item is not applicable/relevant for your study

"consisted of 6-20 sessions of bCBT (involving face-to-face sessions with a therapist and an internet-based program). TAU consisted of usual care for depression."

1b-iii) Open vs. closed, web-based (self-assessment) vs. face-to-face assessments in the METHODS section of the ABSTRACT

Mention how participants were recruited (online vs. offline), e.g., from an open access website or from a clinic or a closed online user group (closed usergroup trial), and clarify if this was a purely web-based trial, or there were face-to-face components (as part of the intervention or for assessment). Clearly say if outcomes were self-assessed through questionnaires (as common in web-based trials). Note: In traditional offline trials, an open trial (open-label trial) is a type of clinical trial in which both the researchers and participants know which treatment is being administered. To avoid confusion, use “blinded” or “unblinded” to indicated the level of blinding instead of “open”, as “open” in web-based trials usually refers to “open access” (i.e. participants can self-enrol). (Note: Only report in the abstract what the main paper is reporting. If this information is missing from the main body of text, consider adding it)

	1	2	3	4	5	
subitem not at all important	○	○	○	○	○	essential

Does your paper address subitem 1b-iii?

Copy and paste relevant sections from the manuscript abstract (include quotes in quotation marks "like this" to indicate direct quotes from your manuscript), or elaborate on this item by providing additional information not in the ms, or briefly explain why the item is not applicable/relevant for your study

Your answer

1b-iv) RESULTS section in abstract must contain use data

Report number of participants enrolled/assessed in each group, the use/uptake of the intervention (e.g., attrition/adherence metrics, use over time, number of logins etc.), in addition to primary/secondary outcomes. (Note: Only report in the abstract what the main paper is reporting. If this information is missing from the main body of text, consider adding it)

	1	2	3	4	5	
subitem not at all important	○	○	○	○	○	essential

Does your paper address subitem 1b-iv?

Copy and paste relevant sections from the manuscript abstract (include quotes in quotation marks "like this" to indicate direct quotes from your manuscript), or elaborate on this item by providing additional information not in the ms, or briefly explain why the item is not applicable/relevant for your study

Your answer

1b-v) CONCLUSIONS/DISCUSSION in abstract for negative trials

Conclusions/Discussions in abstract for negative trials: Discuss the primary outcome - if the trial is negative (primary outcome not changed), and the intervention was not used, discuss whether negative results are attributable to lack of uptake and discuss reasons. (Note: Only report in the abstract what the main paper is reporting. If this information is missing from the main body of text, consider adding it)

	1	2	3	4	5	
subitem not at all important	○	○	○	○	○	essential

Does your paper address subitem 1b-v?

Copy and paste relevant sections from the manuscript abstract (include quotes in quotation marks "like this" to indicate direct quotes from your manuscript), or elaborate on this item by providing additional information not in the ms, or briefly explain why the item is not applicable/relevant for your study

Your answer

INTRODUCTION

2a) In INTRODUCTION: Scientific background and explanation of rationale

2a-i) Problem and the type of system/solution

Describe the problem and the type of system/solution that is object of the study: intended as stand-alone intervention vs. incorporated in broader health care program? Intended for a particular patient population? Goals of the intervention, e.g., being more cost-effective to other interventions, replace or complement other solutions? (Note: Details about the intervention are provided in "Methods" under 5)

	1	2	3	4	5	
subitem not at all important	○	○	○	◉	○	essential

Clear selection

Does your paper address subitem 2a-i? *

Copy and paste relevant sections from the manuscript (include quotes in quotation marks "like this" to indicate direct quotes from your manuscript), or elaborate on this item by providing additional information not in the ms, or briefly explain why the item is not applicable/relevant for your study

"To this end, only 3 studies have examined the working alliance in blended CBT (bCBT)"
"To our knowledge, we report the largest investigation of the working alliance in bCBT for depression, using pooled data from 9 country sites involved in a pragmatic noninferiority randomized controlled trial investigating the effectiveness of bCBT for depression when compared with treatment as usual (TAU)"

2a-ii) Scientific background, rationale: What is known about the (type of) system

Scientific background, rationale: What is known about the (type of) system that is the object of the study (be sure to discuss the use of similar systems for other conditions/diagnoses, if appropiate), motivation for the study, i.e. what are the reasons for and what is the context for this specific study, from which stakeholder viewpoint is the study performed, potential impact of findings [2]. Briefly justify the choice of the comparator.

	1	2	3	4	5	
subitem not at all important	○	○	○	◉	○	essential

Clear selection

Does your paper address subitem 2a-ii? *

Copy and paste relevant sections from the manuscript (include quotes in quotation marks "like this" to indicate direct quotes from your manuscript), or elaborate on this item by providing additional information not in the ms, or briefly explain why the item is not applicable/relevant for your study

"Systematic reviews that have addressed these questions specifically in relation to interventions that are guided, adopt CBT [21], or target the treatment of depression [27] found that the working alliance can be established in guided DMHIs at a comparable level to face-to-face therapy [21]; however, the literature on the outcome-alliance relationship is mixed [21,26,27]."

"To this end, only 3 studies have examined the working alliance in blended CBT (bCBT)."

2b) In INTRODUCTION: Specific objectives or hypotheses

Does your paper address CONSORT subitem 2b? *

Copy and paste relevant sections from the manuscript (include quotes in quotation marks "like this" to indicate direct quotes from your manuscript), or elaborate on this item by providing additional information not in the ms, or briefly explain why the item is not applicable/relevant for your study

"Our primary objectives are to test the difference in working alliance scores between bCBT and TAU (objective 1), and determine if working alliance scores are associated with depression scores (objective 2). Our secondary objective is to test for an interaction between system usability and the working alliance with regard to an association between the working alliance and depression scores in bCBT (objective3)."

METHODS

3a) Description of trial design (such as parallel, factorial) including allocation ratio

Does your paper address CONSORT subitem 3a? *

Copy and paste relevant sections from the manuscript (include quotes in quotation marks "like this" to indicate direct quotes from your manuscript), or elaborate on this item by providing additional information not in the ms, or briefly explain why the item is not applicable/relevant for your study

"We conducted a nonprespecified secondary analysis of data collected in the E-COMPARED study, a large European 2-arm, noninferiority randomized controlled trial investigating the effectiveness of bCBT compared with TAU across 9 European countries"

3b) Important changes to methods after trial commencement (such as eligibility criteria), with reasons

Does your paper address CONSORT subitem 3b? *

Copy and paste relevant sections from the manuscript (include quotes in quotation marks "like this" to indicate direct quotes from your manuscript), or elaborate on this item by providing additional information not in the ms, or briefly explain why the item is not applicable/relevant for your study

The only significant change was the addition of new recruitment site, indicated as follows – "Following the start of recruitment, an additional satellite site was added in Denmark to boost recruitment [38]".

3b-i) Bug fixes, Downtimes, Content Changes

Bug fixes, Downtimes, Content Changes: ehealth systems are often dynamic systems. A description of changes to methods therefore also includes important changes made on the intervention or comparator during the trial (e.g., major bug fixes or changes in the functionality or content) (5-iii) and other "unexpected events" that may have influenced study design such as staff changes, system failures/downtimes, etc. [2].

	1	2	3	4	5	
subitem not at all important	○	○	○	○	○	essential

Does your paper address subitem 3b-i?

Copy and paste relevant sections from the manuscript (include quotes in quotation marks "like this" to indicate direct quotes from your manuscript), or elaborate on this item by providing additional information not in the ms, or briefly explain why the item is not applicable/relevant for your study

Your answer

4a) Eligibility criteria for participants

Does your paper address CONSORT subitem 4a? *

Copy and paste relevant sections from the manuscript (include quotes in quotation marks "like this" to indicate direct quotes from your manuscript), or elaborate on this item by providing additional information not in the ms, or briefly explain why the item is not applicable/relevant for your study

"The inclusion criteria applied at all sites were as follows: age ≥18 years and meeting the diagnostic criteria for major depressive disorder as confirmed by the MINI International Neuropsychiatric Interview (M.I.N.I) version 5.0 [40]. The exclusion criteria were as follows: high risk of suicide and psychiatric comorbidity (ie, substance dependence, bipolar affective disorder, psychotic illness, or obsessive compulsive disorder) assessed during the M.I.N.I. interview; receiving psychological treatment for depression in primary or specialized mental health care at the point of recruitment; inability to comprehend the spoken and written language of the country site; lacking access to a computer or a fast internet connection (ie, broadband or comparable); and lacking a smartphone or being unwilling to carry a smartphone if one was provided by the research team [35]."

4a-i) Computer / Internet literacy

Computer / Internet literacy is often an implicit “de facto” eligibility criterion - this should be explicitly clarified.

	1	2	3	4	5	
subitem not at all important	○	○	○	○	○	essential

Does your paper address subitem 4a-i?

Copy and paste relevant sections from the manuscript (include quotes in quotation marks "like this" to indicate direct quotes from your manuscript), or elaborate on this item by providing additional information not in the ms, or briefly explain why the item is not applicable/relevant for your study

Your answer

4a-ii) Open vs. closed, web-based vs. face-to-face assessments:

Open vs. closed, web-based vs. face-to-face assessments: Mention how participants were recruited (online vs. offline), e.g., from an open access website or from a clinic, and clarify if this was a purely web-based trial, or there were face-to-face components (as part of the intervention or for assessment), i.e., to what degree got the study team to know the participant. In online-only trials, clarify if participants were quasi-anonymous and whether having multiple identities was possible or whether technical or logistical measures (e.g., cookies, email confirmation, phone calls) were used to detect/prevent these.

	1	2	3	4	5	
subitem not at all important	○	○	○	○	○	essential

Does your paper address subitem 4a-ii? *

Copy and paste relevant sections from the manuscript (include quotes in quotation marks "like this" to indicate direct quotes from your manuscript), or elaborate on this item by providing additional information not in the ms, or briefly explain why the item is not applicable/relevant for your study

"Clients seeking treatment for depression were recruited, assessed, and treated across routine primary care in Germany, Poland, Spain, Sweden, and the United Kingdom, and specialized mental health services in France, the Netherlands, Switzerland, and Denmark [35]. Following the start of recruitment, an additional satellite site was added in Denmark to boost recruitment [38]"

"Recruitment procedures differed in each country, but all sites screened new clients seeking help for depression, who scored 5 or higher on the Patient Health Questionnaire-9 (PHQ-9)"

4a-iii) Information giving during recruitment

Information given during recruitment. Specify how participants were briefed for recruitment and in the informed consent procedures (e.g., publish the informed consent documentation as appendix, see also item X26), as this information may have an effect on user self-selection, user expectation and may also bias results.

	1	2	3	4	5	
subitem not at all important	○	○	○	○	○	essential

Does your paper address subitem 4a-iii?

Copy and paste relevant sections from the manuscript (include quotes in quotation marks "like this" to indicate direct quotes from your manuscript), or elaborate on this item by providing additional information not in the ms, or briefly explain why the item is not applicable/relevant for your study

Your answer

4b) Settings and locations where the data were collected

Does your paper address CONSORT subitem 4b? *

Copy and paste relevant sections from the manuscript (include quotes in quotation marks "like this" to indicate direct quotes from your manuscript), or elaborate on this item by providing additional information not in the ms, or briefly explain why the item is not applicable/relevant for your study

"Clients seeking treatment for depression were recruited, assessed, and treated across routine primary care in Germany, Poland, Spain, Sweden, and the United Kingdom, and specialized mental health services in France, the Netherlands, Switzerland, and Denmark [35]. Following the start of recruitment, an additional satellite site was added in Denmark to boost recruitment [38]"

4b-i) Report if outcomes were (self-)assessed through online questionnaires

Clearly report if outcomes were (self-)assessed through online questionnaires (as common in web-based trials) or otherwise.

	1	2	3	4	5	
subitem not at all important	○	○	○	○	○	essential

Does your paper address subitem 4b-i? *

Copy and paste relevant sections from the manuscript (include quotes in quotation marks "like this" to indicate direct quotes from your manuscript), or elaborate on this item by providing additional information not in the ms, or briefly explain why the item is not applicable/relevant for your study

"Baseline data were completed online, face-to-face, via telephone, or a combination of these approaches. The 3-month follow-up assessments were largely completed online, with the exception of the PHQ-9 that was collected via telephone to maximize data collection of the trial's primary outcome. Data that were directly collected by researchers (ie, either in person or via telephone) were double entered to increase the accuracy of the data entry process."

4b-ii) Report how institutional affiliations are displayed

Report how institutional affiliations are displayed to potential participants [on ehealth media], as affiliations with prestigious hospitals or universities may affect volunteer rates, use, and reactions with regards to an intervention.(Not a required item – describe only if this may bias results)

	1	2	3	4	5	
subitem not at all important	○	○	○	○	○	essential

Does your paper address subitem 4b-ii?

Copy and paste relevant sections from the manuscript (include quotes in quotation marks "like this" to indicate direct quotes from your manuscript), or elaborate on this item by providing additional information not in the ms, or briefly explain why the item is not applicable/relevant for your study

Your answer

5) The interventions for each group with sufficient details to allow replication, including how and when they were actually administered

5-i) Mention names, credential, affiliations of the developers, sponsors, and owners

Mention names, credential, affiliations of the developers, sponsors, and owners [6] (if authors/evaluators are owners or developer of the software, this needs to be declared in a “Conflict of interest” section or mentioned elsewhere in the manuscript).

	1	2	3	4	5	
subitem not at all important	○	○	○	○	○	essential

Does your paper address subitem 5-i?

Copy and paste relevant sections from the manuscript (include quotes in quotation marks "like this" to indicate direct quotes from your manuscript), or elaborate on this item by providing additional information not in the ms, or briefly explain why the item is not applicable/relevant for your study

Your answer

5-ii) Describe the history/development process

Describe the history/development process of the application and previous formative evaluations (e.g., focus groups, usability testing), as these will have an impact on adoption/use rates and help with interpreting results.

	1	2	3	4	5	
subitem not at all important	○	○	○	○	○	essential

Does your paper address subitem 5-ii?

Copy and paste relevant sections from the manuscript (include quotes in quotation marks "like this" to indicate direct quotes from your manuscript), or elaborate on this item by providing additional information not in the ms, or briefly explain why the item is not applicable/relevant for your study

Your answer

5-iii) Revisions and updating

Revisions and updating. Clearly mention the date and/or version number of the application/intervention (and comparator, if applicable) evaluated, or describe whether the intervention underwent major changes during the evaluation process, or whether the development and/or content was “frozen” during the trial. Describe dynamic components such as news feeds or changing content which may have an impact on the replicability of the intervention (for unexpected events see item 3b).

	1	2	3	4	5	
subitem not at all important	○	○	○	○	○	essential

Does your paper address subitem 5-iii?

Copy and paste relevant sections from the manuscript (include quotes in quotation marks "like this" to indicate direct quotes from your manuscript), or elaborate on this item by providing additional information not in the ms, or briefly explain why the item is not applicable/relevant for your study

Your answer

5-iv) Quality assurance methods

Provide information on quality assurance methods to ensure accuracy and quality of information provided [1], if applicable.

	1	2	3	4	5	
subitem not at all important	○	○	○	○	○	essential

Does your paper address subitem 5-iv?

Copy and paste relevant sections from the manuscript (include quotes in quotation marks "like this" to indicate direct quotes from your manuscript), or elaborate on this item by providing additional information not in the ms, or briefly explain why the item is not applicable/relevant for your study

Your answer

5-v) Ensure replicability by publishing the source code, and/or providing screenshots/screen-capture video, and/or providing flowcharts of the algorithms used

Ensure replicability by publishing the source code, and/or providing screenshots/screen-capture video, and/or providing flowcharts of the algorithms used. Replicability (i.e., other researchers should in principle be able to replicate the study) is a hallmark of scientific reporting.

	1	2	3	4	5	
subitem not at all important	○	○	○	○	○	essential

Does your paper address subitem 5-v?

Copy and paste relevant sections from the manuscript (include quotes in quotation marks "like this" to indicate direct quotes from your manuscript), or elaborate on this item by providing additional information not in the ms, or briefly explain why the item is not applicable/relevant for your study

Your answer

5-vi) Digital preservation

Digital preservation: Provide the URL of the application, but as the intervention is likely to change or disappear over the course of the years; also make sure the intervention is archived (Internet Archive, webcitation.org, and/or publishing the source code or screenshots/videos alongside the article). As pages behind login screens cannot be archived, consider creating demo pages which are accessible without login.

	1	2	3	4	5	
subitem not at all important	○	○	○	○	○	essential

Does your paper address subitem 5-vi?

Copy and paste relevant sections from the manuscript (include quotes in quotation marks "like this" to indicate direct quotes from your manuscript), or elaborate on this item by providing additional information not in the ms, or briefly explain why the item is not applicable/relevant for your study

Your answer

5-vii) Access

Access: Describe how participants accessed the application, in what setting/context, if they had to pay (or were paid) or not, whether they had to be a member of specific group. If known, describe how participants obtained "access to the platform and Internet" [1]. To ensure access for editors/reviewers/readers, consider to provide a "backdoor" login account or demo mode for reviewers/readers to explore the application (also important for archiving purposes, see vi).

	1	2	3	4	5	
subitem not at all important	○	○	○	○	○	essential

Does your paper address subitem 5-vii? *

Copy and paste relevant sections from the manuscript (include quotes in quotation marks "like this" to indicate direct quotes from your manuscript), or elaborate on this item by providing additional information not in the ms, or briefly explain why the item is not applicable/relevant for your study

Participants gained access through their participation on trial, following their access to services that the trial recruited from.

5-viii) Mode of delivery, features/functionalities/components of the intervention and comparator, and the theoretical framework

Describe mode of delivery, features/functionalities/components of the intervention and comparator, and the theoretical framework [6] used to design them (instructional strategy [1], behaviour change techniques, persuasive features, etc., see e.g., [7, 8] for terminology). This includes an in-depth description of the content (including where it is coming from and who developed it) [1]," whether [and how] it is tailored to individual circumstances and allows users to track their progress and receive feedback" [6]. This also includes a description of communication delivery channels and – if computer-mediated communication is a component – whether communication was synchronous or asynchronous [6]. It also includes information on presentation strategies [1], including page design principles, average amount of text on pages, presence of hyperlinks to other resources, etc. [1].

	1	2	3	4	5	
subitem not at all important	○	○	○	○	○	essential

Does your paper address subitem 5-viii? *

Copy and paste relevant sections from the manuscript (include quotes in quotation marks "like this" to indicate direct quotes from your manuscript), or elaborate on this item by providing additional information not in the ms, or briefly explain why the item is not applicable/relevant for your study

"bCBT for depression consisted of integrating a digital program (iCBT plus mobile app) with face-to-face CBT in a single treatment protocol [35,41]. iCBT programs included 4cmandatory core modules of CBT (ie, psychoeducation, behavioral activation, cognitive restructuring, and relapse prevention) plus optional modules (eg, physical exercise and problem solving) typically completed at home, while face-to-face CBT was delivered in the clinic [35]. Clients worked through treatment modules, completed exercises, and monitored their symptoms on the digital program, while face-to-face sessions were used by the therapist to set up modules, monitor client progress, and address client-specific needs. Sequencing and time spent on each module were flexibly applied; however, the 4 mandatory modules on the digital program had to be completed. Data on treatment and dosage were not collected for TAU in the trial. See Table 1 for a breakdown of recruitment, bCBT format and dosage, and treatments offered in TAU across all country sites [30,35,42]."

"Based on the registered data, 194 therapists delivered trial interventions. In Germany, therapists only delivered bCBT in the treatment arm, whereas therapists from the remaining 8 country sites delivered interventions across both treatment arms. The risk of contamination was not perceived as a concern, as CBT was also offered in TAU, with the focus of the trial on investigating the blending of an internet-based intervention with face-to-face CBT when compared to routine care. Data on therapist ratings of the working alliance will be published in a separate paper to enable comprehensive reporting and discussion of the findings."

5-ix) Describe use parameters

Describe use parameters (e.g., intended "doses" and optimal timing for use). Clarify what instructions or recommendations were given to the user, e.g., regarding timing, frequency, heaviness of use, if any, or was the intervention used ad libitum.

	1	2	3	4	5	
subitem not at all important	○	○	○	○	○	essential

Does your paper address subitem 5-ix?

Copy and paste relevant sections from the manuscript (include quotes in quotation marks "like this" to indicate direct quotes from your manuscript), or elaborate on this item by providing additional information not in the ms, or briefly explain why the item is not applicable/relevant for your study

Your answer

5-x) Clarify the level of human involvement

Clarify the level of human involvement (care providers or health professionals, also technical assistance) in the e-intervention or as co-intervention (detail number and expertise of professionals involved, if any, as well as “type of assistance offered, the timing and frequency of the support, how it is initiated, and the medium by which the assistance is delivered”. It may be necessary to distinguish between the level of human involvement required for the trial, and the level of human involvement required for a routine application outside of a RCT setting (discuss under item 21 – generalizability).

	1	2	3	4	5	
subitem not at all important	○	○	○	○	○	essential

Does your paper address subitem 5-x?

Copy and paste relevant sections from the manuscript (include quotes in quotation marks "like this" to indicate direct quotes from your manuscript), or elaborate on this item by providing additional information not in the ms, or briefly explain why the item is not applicable/relevant for your study

Your answer

5-xi) Report any prompts/reminders used

Report any prompts/reminders used: Clarify if there were prompts (letters, emails, phone calls, SMS) to use the application, what triggered them, frequency etc. It may be necessary to distinguish between the level of prompts/reminders required for the trial, and the level of prompts/reminders for a routine application outside of a RCT setting (discuss under item 21 – generalizability).

	1	2	3	4	5	
subitem not at all important	○	○	○	○	○	essential

Does your paper address subitem 5-xi? *

Copy and paste relevant sections from the manuscript (include quotes in quotation marks "like this" to indicate direct quotes from your manuscript), or elaborate on this item by providing additional information not in the ms, or briefly explain why the item is not applicable/relevant for your study

No routine prompts were made to encourage the participants/end-users to use the iCBT intervention, from the trial team. As the intervention was blended, therapists were required to monitor participants use of the iCBT program and remind participants during their face-to-face sessions, or between sessions either online or by telephone. However, this was not protocolized and was based on the therapist's discretion. Moreover, data on the number of prompts directed by participants by the therapist were not systematically collected on the trial.

5-xii) Describe any co-interventions (incl. training/support)

Describe any co-interventions (incl. training/support): Clearly state any interventions that are provided in addition to the targeted eHealth intervention, as ehealth intervention may not be designed as stand-alone intervention. This includes training sessions and support [1]. It may be necessary to distinguish between the level of training required for the trial, and the level of training for a routine application outside of a RCT setting (discuss under item 21 – generalizability.

	1	2	3	4	5	
subitem not at all important	○	○	○	○	○	essential

Does your paper address subitem 5-xii? *

Copy and paste relevant sections from the manuscript (include quotes in quotation marks "like this" to indicate direct quotes from your manuscript), or elaborate on this item by providing additional information not in the ms, or briefly explain why the item is not applicable/relevant for your study

Therapists on the trial were provided with an induction to the study (including aims, objectives and question(s) being addressed by the trial) and were provided with training on the digital intervention if the online programs were not adopted by the services recruited from before the trial.

6a) Completely defined pre-specified primary and secondary outcome measures, including how and when they were assessed

Does your paper address CONSORT subitem 6a? *

Copy and paste relevant sections from the manuscript (include quotes in quotation marks "like this" to indicate direct quotes from your manuscript), or elaborate on this item by providing additional information not in the ms, or briefly explain why the item is not applicable/relevant for your study

Primary outcomes
"The study outcomes were the working alliance and depression severity, which were measured using the Working Alliance Inventory-Short Revised–Client (WAI-SR-C) [46] and the PHQ-9 [39], respectively. The WAI-SR-C scale is based on the theory of working alliance containing 3-item subscales assessing bond, task, and goals by Bordin [15,16]. The 12 items are rated on a 5-point scale from 1 (seldom) to 5 (always), with total scores ranging between 12 and 60. Higher scores on the scale indicate better working alliance. The WAI-SR-C scale has demonstrated good reliability (internal consistency) for all 3 factors, including the bond, task, and goals subscales (Cronbach α=0.92, 0.92, and 0.89, respectively) [47]. The scale has been shown to be correlated with other therapeutic alliance scales such as the California Therapeutic Alliance Rating System [19,48] and the Helping Alliance Questionnaire-II [19,49]. The WAI-SR-C scale was only administered at 3-month assessments. Data for the WAI-SR-C scale were not collected in the TAU arm of the Swedish country site.

The PHQ-9 [39] was used to assess depression as the trial’s primary clinical outcome. The PHQ-9 is a 9-item scale that can be used to screen and diagnose people for depressive disorders. Each of the 9 items is scored on a 4-point scale from 0 (not at all) to 3 (nearly every day). The total score ranges between 0 and 27, with higher scores indicating greater symptom severity. Depression severity can be grouped into the following: mild (score 0-5), moderate (6-10), moderately severe (11-15), and severe (≥16). The PHQ-9 has been shown to have good psychometric properties [39] and has demonstrated its utility as a valid diagnostic tool [50]. The PHQ-9 was administered at the baseline and 3-, 6-, and 12-month assessments; however this study only used baseline and 3-month assessment data as the study was interested in investigating depression scores."

Secondary outcomes
"System Usability Scale-Client (SUS-C) [51,52] was used to assess the usability of the digital programs. The scale is a 10-item self-reported questionnaire. Items are measured on a 5-point scale ranging from 1 (strongly disagree) to 5 (strongly agree). The total SUS-C score ranges between 10 and 50 to produce a global score. Higher scores indicate better system usability. The total sum score has been found to be a valid and interpretable measure to assess the usability of internet-based interventions by professionals in mental health care settings [53]. The SUS showed high internal reliability (eg, coefficient Ω=0.91) and good concurrent validity and sensitivity [52,53]. The SUS-C was administered at the 3-month follow-up assessment."

Demographic Data
"Demographic data on the participant’s gender, age, educational attainment, marital status, and country site were collected at baseline. Baseline variables that were used as covariates in the regression models included age, gender (male, female, and other), marital status (single, divorced, widowed, living together, and married), and educational level (low, middle, and high, corresponding to secondary school education or equivalent [low], college or equivalent [middle], and university degree or higher [high])."

6a-i) Online questionnaires: describe if they were validated for online use and apply CHERRIES items to describe how the questionnaires were designed/deployed

If outcomes were obtained through online questionnaires, describe if they were validated for online use and apply CHERRIES items to describe how the questionnaires were designed/deployed [9].

	1	2	3	4	5	
subitem not at all important	○	○	○	○	○	essential

Does your paper address subitem 6a-i?

Copy and paste relevant sections from manuscript text

Your answer

6a-ii) Describe whether and how “use” (including intensity of use/dosage) was defined/measured/monitored

Describe whether and how “use” (including intensity of use/dosage) was defined/measured/monitored (logins, logfile analysis, etc.). Use/adoption metrics are important process outcomes that should be reported in any ehealth trial.

	1	2	3	4	5	
subitem not at all important	○	○	○	○	○	essential

Does your paper address subitem 6a-ii?

Copy and paste relevant sections from manuscript text

Your answer

6a-iii) Describe whether, how, and when qualitative feedback from participants was obtained

Describe whether, how, and when qualitative feedback from participants was obtained (e.g., through emails, feedback forms, interviews, focus groups).

	1	2	3	4	5	
subitem not at all important	○	○	○	○	○	essential

Does your paper address subitem 6a-iii?

Copy and paste relevant sections from manuscript text

Your answer

6b) Any changes to trial outcomes after the trial commenced, with reasons

Does your paper address CONSORT subitem 6b? *

Copy and paste relevant sections from the manuscript (include quotes in quotation marks "like this" to indicate direct quotes from your manuscript), or elaborate on this item by providing additional information not in the ms, or briefly explain why the item is not applicable/relevant for your study

There were no changes to the trial's outcome measures. It should be noted that this study conducted a secondary analysis of the trial's secondary outcomes, and not the primary outcomes of the trial, which will be published in a separate paper.

7a) How sample size was determined

NPT: When applicable, details of whether and how the clustering by care provides or centers was addressed

7a-i) Describe whether and how expected attrition was taken into account when calculating the sample size

Describe whether and how expected attrition was taken into account when calculating the sample size.

	1	2	3	4	5	
subitem not at all important	○	○	○	○	○	essential

Does your paper address subitem 7a-i?

Copy and paste relevant sections from manuscript title (include quotes in quotation marks "like this" to indicate direct quotes from your manuscript), or elaborate on this item by providing additional information not in the ms, or briefly explain why the item is not applicable/relevant for your study

This study is a secondary data analysis, therefore a sample size calculation was not required.

7b) When applicable, explanation of any interim analyses and stopping guidelines

Does your paper address CONSORT subitem 7b? *

Copy and paste relevant sections from the manuscript (include quotes in quotation marks "like this" to indicate direct quotes from your manuscript), or elaborate on this item by providing additional information not in the ms, or briefly explain why the item is not applicable/relevant for your study

Interim analyses and stopping guidelines were not applicable to this secondary data analysis.

8a) Method used to generate the random allocation sequence

NPT: When applicable, how care providers were allocated to each trial group

Does your paper address CONSORT subitem 8a? *

Copy and paste relevant sections from the manuscript (include quotes in quotation marks "like this" to indicate direct quotes from your manuscript), or elaborate on this item by providing additional information not in the ms, or briefly explain why the item is not applicable/relevant for your study

"...participants were randomized to 1 of 2 treatment arms (bCBT or TAU) using block randomization, with stratification by country [35]. All participants provided written informed consent before taking part in the trial [35]."

8b) Type of randomisation; details of any restriction (such as blocking and block size)

Does your paper address CONSORT subitem 8b? *

Copy and paste relevant sections from the manuscript (include quotes in quotation marks "like this" to indicate direct quotes from your manuscript), or elaborate on this item by providing additional information not in the ms, or briefly explain why the item is not applicable/relevant for your study

"Randomization will be conducted centrally by the (PI) organization (VU Amsterdam) by a team of independent researchers (the randomization team). The randomization team allocates patients to treatment in all countries except for France where the allocation process will be automated and the UK where the allocation process will be outsourced to a local independent researcher as for logistical reasons the randomization outcome should be instantly available. Randomization will take place at an individual level, stratified by country, after the eligibility and baseline assessment. Further stratification on recruitment location will be applied in France (11 centers), Switzerland (3 centers), The Netherlands (3 centers), and Sweden (two cities). The randomization team will create the allocation scheme with a computerized random number generator (Random Allocation Software) at an allocation ratio of 1:1. Block randomization will be used with variable block sizes that vary between 8 and 14 allocations per block (in France block sizes of 2 and 4 will be applied because of the large number of strata). Subjects will be randomized into two groups: bCBT or TAU. All investigators and clinicians will be unknown to the randomization scheme. Blinding for treatment allocation is not possible as it will be clear to both therapists and patients whether the treatment is blended or not. However, outcome assessors will be blinded."

Information above was copied from the trial protocol:
Kleiboer, A., Smit, J., Bosmans, J. et al. European COMPARative Effectiveness research on blended Depression treatment versus treatment-as-usual (E-COMPARED): study protocol for a randomized controlled, non-inferiority trial in eight European countries. Trials 17, 387 (2016). https://doi.org/10.1186/s13063-016-1511-1

9) Mechanism used to implement the random allocation sequence (such as sequentially numbered containers), describing any steps taken to conceal the sequence until interventions were assigned

Does your paper address CONSORT subitem 9? *

Copy and paste relevant sections from the manuscript (include quotes in quotation marks "like this" to indicate direct quotes from your manuscript), or elaborate on this item by providing additional information not in the ms, or briefly explain why the item is not applicable/relevant for your study

"Block randomization will be used with variable block sizes that vary between 8 and 14 allocations per block (in France block sizes of 2 and 4 will be applied because of the large number of strata). Subjects will be randomized into two groups: bCBT or TAU. All investigators and clinicians will be unknown to the randomization scheme. Blinding for treatment allocation is not possible as it will be clear to both therapists and patients whether the treatment is blended or not. However, outcome assessors will be blinded."

Information above was copied from the trial protocol:
Kleiboer, A., Smit, J., Bosmans, J. et al. European COMPARative Effectiveness research on blended Depression treatment versus treatment-as-usual (E-COMPARED): study protocol for a randomized controlled, non-inferiority trial in eight European countries. Trials 17, 387 (2016). https://doi.org/10.1186/s13063-016-1511-1

10) Who generated the random allocation sequence, who enrolled participants, and who assigned participants to interventions

Does your paper address CONSORT subitem 10? *

Copy and paste relevant sections from the manuscript (include quotes in quotation marks "like this" to indicate direct quotes from your manuscript), or elaborate on this item by providing additional information not in the ms, or briefly explain why the item is not applicable/relevant for your study

"Randomization will be conducted centrally by the (PI) organization (VU Amsterdam) by a team of independent researchers (the randomization team).The randomization team allocates patients to treatment in all countries except for France where the allocation process will be automated and the UK where the allocation process will be outsourced to a local independent researcher as for logistical reasons the randomization outcome should be instantly available. Randomization will take place at an individual level, stratified by country, after the eligibility and baseline assessment. Further stratification on recruitment location will be applied in France (11 centers), Switzerland (3 centers), The Netherlands (3 centers), and Sweden (two cities)."

Information above was copied from the trial protocol:
Kleiboer, A., Smit, J., Bosmans, J. et al. European COMPARative Effectiveness research on blended Depression treatment versus treatment-as-usual (E-COMPARED): study protocol for a randomized controlled, non-inferiority trial in eight European countries. Trials 17, 387 (2016). https://doi.org/10.1186/s13063-016-1511-1

11a) If done, who was blinded after assignment to interventions (for example, participants, care providers, those assessing outcomes) and how

NPT: Whether or not administering co-interventions were blinded to group assignment

11a-i) Specify who was blinded, and who wasn’t

Specify who was blinded, and who wasn’t. Usually, in web-based trials it is not possible to blind the participants [1, 3] (this should be clearly acknowledged), but it may be possible to blind outcome assessors, those doing data analysis or those administering co-interventions (if any).

	1	2	3	4	5	
subitem not at all important	○	○	○	○	○	essential

Does your paper address subitem 11a-i? *

Copy and paste relevant sections from the manuscript (include quotes in quotation marks "like this" to indicate direct quotes from your manuscript), or elaborate on this item by providing additional information not in the ms, or briefly explain why the item is not applicable/relevant for your study

"Blinding for treatment allocation is not possible as it will be clear to both therapists and patients whether the treatment is blended or not. However, outcome assessors will be blinded."

]Information above was copied from the trial protocol:
Kleiboer, A., Smit, J., Bosmans, J. et al. European COMPARative Effectiveness research on blended Depression treatment versus treatment-as-usual (E-COMPARED): study protocol for a randomized controlled, non-inferiority trial in eight European countries. Trials 17, 387 (2016). https://doi.org/10.1186/s13063-016-1511-1

11a-ii) Discuss e.g., whether participants knew which intervention was the “intervention of interest” and which one was the “comparator”

Informed consent procedures (4a-ii) can create biases and certain expectations - discuss e.g., whether participants knew which intervention was the “intervention of interest” and which one was the “comparator”.

	1	2	3	4	5	
subitem not at all important	○	○	○	○	○	essential

Does your paper address subitem 11a-ii?

Copy and paste relevant sections from the manuscript (include quotes in quotation marks "like this" to indicate direct quotes from your manuscript), or elaborate on this item by providing additional information not in the ms, or briefly explain why the item is not applicable/relevant for your study

Your answer

11b) If relevant, description of the similarity of interventions
(this item is usually not relevant for ehealth trials as it refers to similarity of a placebo or sham intervention to a active medication/intervention)

Does your paper address CONSORT subitem 11b? *

Copy and paste relevant sections from the manuscript (include quotes in quotation marks "like this" to indicate direct quotes from your manuscript), or elaborate on this item by providing additional information not in the ms, or briefly explain why the item is not applicable/relevant for your study

TAU for many of the countries also offered talking therapies such as CBT, although this was only delivered in person and not online.

12a) Statistical methods used to compare groups for primary and secondary outcomes
NPT: When applicable, details of whether and how the clustering by care providers or centers was addressed

Does your paper address CONSORT subitem 12a? *

Copy and paste relevant sections from the manuscript (include quotes in quotation marks "like this" to indicate direct quotes from your manuscript), or elaborate on this item by providing additional information not in the ms, or briefly explain why the item is not applicable/relevant for your study

Regarding the secondary analysis conducted in this study: “In order to test if treatment assignment predicted WAI-SR-C scores at 3-month assessments (objective 1), a fixed effects linear regression model [58] was fitted separately for WAI-SR-C composite and subscale scores (goals, task, and bond). Four models were fitted altogether.”

All models adjusted for country-sites.

12a-i) Imputation techniques to deal with attrition / missing values

Imputation techniques to deal with attrition / missing values: Not all participants will use the intervention/comparator as intended and attrition is typically high in ehealth trials. Specify how participants who did not use the application or dropped out from the trial were treated in the statistical analysis (a complete case analysis is strongly discouraged, and simple imputation techniques such as LOCF may also be problematic [4]).

	1	2	3	4	5	
subitem not at all important	○	○	○	○	○	essential

Does your paper address subitem 12a-i? *

Copy and paste relevant sections from the manuscript (include quotes in quotation marks "like this" to indicate direct quotes from your manuscript), or elaborate on this item by providing additional information not in the ms, or briefly explain why the item is not applicable/relevant for your study

"We imputed data sets using the chained equation approach [59]"

"In the imputation model, we included all variables that were part of the analyses, including observations from the PHQ-9 at baseline and demographic variables. To account for the interaction term in the regression model, data were imputed using the just another variable (JAV) approach [60]. Multiple imputation was performed separately for bCBT and TAU to allow for condition-specific variables to be considered. For example, the SUS-C variable was only entered in the bCBT arm, as those in the TAU arm did not use a digital program."

12b) Methods for additional analyses, such as subgroup analyses and adjusted analyses

Does your paper address CONSORT subitem 12b? *

Copy and paste relevant sections from the manuscript (include quotes in quotation marks "like this" to indicate direct quotes from your manuscript), or elaborate on this item by providing additional information not in the ms, or briefly explain why the item is not applicable/relevant for your study

"Post-hoc sensitivity analyses were conducted to examine if the multiple imputation approach that was used to handle missing data would lead to different conclusions when compared to a complete case analysis. Under the MAR assumption, consistent findings between the primary analysis and sensitivity analysis can strengthen the reliability of the findings [61-64], at least in situations where both the primary and sensitivity analyses are expected to be valid under similar assumptions (eg, multiple imputation and complete case analysis under the MAR assumption in the outcome variable only). Owing to the heterogeneity of interventions offered in the TAU arm within the current pragmatic trial, a subgroup analysis was conducted to explore the magnitude of treatment effects on the working alliance when using a subset of the sample, which compared bCBT with face-to-face CBT offered in the TAU arm in Denmark, France, Poland, Switzerland, and the United Kingdom country sites of the E-COMPARED trial [35]. The subanalysis replicated the main analysis in just 5 country sites. This enabled the working alliance in bCBT to be directly compared with a defined comparator. Results between the primary analysis and the subanalysis were compared to understand if results vary when there are multiple interventions in TAU and when there is a defined comparator (ie, face-to-face CBT) [65-67]."

X26) REB/IRB Approval and Ethical Considerations [recommended as subheading under "Methods"] (not a CONSORT item)

X26-i) Comment on ethics committee approval

	1	2	3	4	5	
subitem not at all important	○	○	○	○	○	essential

Does your paper address subitem X26-i?

Copy and paste relevant sections from the manuscript (include quotes in quotation marks "like this" to indicate direct quotes from your manuscript), or elaborate on this item by providing additional information not in the ms, or briefly explain why the item is not applicable/relevant for your study

Your answer

x26-ii) Outline informed consent procedures

Outline informed consent procedures e.g., if consent was obtained offline or online (how? Checkbox, etc.?), and what information was provided (see 4a-ii). See [6] for some items to be included in informed consent documents.

	1	2	3	4	5	
subitem not at all important	○	○	○	○	○	essential

Does your paper address subitem X26-ii?

Copy and paste relevant sections from the manuscript (include quotes in quotation marks "like this" to indicate direct quotes from your manuscript), or elaborate on this item by providing additional information not in the ms, or briefly explain why the item is not applicable/relevant for your study

Your answer

X26-iii) Safety and security procedures

Safety and security procedures, incl. privacy considerations, and any steps taken to reduce the likelihood or detection of harm (e.g., education and training, availability of a hotline)

	1	2	3	4	5	
subitem not at all important	○	○	○	○	○	essential

Does your paper address subitem X26-iii?

Copy and paste relevant sections from the manuscript (include quotes in quotation marks "like this" to indicate direct quotes from your manuscript), or elaborate on this item by providing additional information not in the ms, or briefly explain why the item is not applicable/relevant for your study

Your answer

RESULTS

13a) For each group, the numbers of participants who were randomly assigned, received intended treatment, and were analysed for the primary outcome

NPT: The number of care providers or centers performing the intervention in each group and the number of patients treated by each care provider in each center

Does your paper address CONSORT subitem 13a? *

Copy and paste relevant sections from the manuscript (include quotes in quotation marks "like this" to indicate direct quotes from your manuscript), or elaborate on this item by providing additional information not in the ms, or briefly explain why the item is not applicable/relevant for your study

"Among the 943 participants who consented and were randomized in the trial (bCBT=476; TAU=467)."

13b) For each group, losses and exclusions after randomisation, together with reasons

Does your paper address CONSORT subitem 13b? (NOTE: Preferably, this is shown in a CONSORT flow diagram) *

Copy and paste relevant sections from the manuscript (include quotes in quotation marks "like this" to indicate direct quotes from your manuscript), or elaborate on this item by providing additional information not in the ms, or briefly explain why the item is not applicable/relevant for your study

Client rated working alliance losses at 3 month assessments (b-CBT=n=141; TAU= n=335)

13b-i) Attrition diagram

Strongly recommended: An attrition diagram (e.g., proportion of participants still logging in or using the intervention/comparator in each group plotted over time, similar to a survival curve) or other figures or tables demonstrating usage/dose/engagement.

	1	2	3	4	5	
subitem not at all important	○	○	○	○	○	essential

Does your paper address subitem 13b-i?

Copy and paste relevant sections from the manuscript or cite the figure number if applicable (include quotes in quotation marks "like this" to indicate direct quotes from your manuscript), or elaborate on this item by providing additional information not in the ms, or briefly explain why the item is not applicable/relevant for your study

Your answer

14a) Dates defining the periods of recruitment and follow-up

Does your paper address CONSORT subitem 14a? *

Copy and paste relevant sections from the manuscript (include quotes in quotation marks "like this" to indicate direct quotes from your manuscript), or elaborate on this item by providing additional information not in the ms, or briefly explain why the item is not applicable/relevant for your study

"Data were collected between April 2015 and December 2017"

Data for the working alliance was only collected 3-months after baseline assessments. Depression scores were collected at 3-months, 6-months and 12-months after baseline assessments for depression. However, this study only used data for baseline and 3-month follow-up assessments.

14a-i) Indicate if critical "secular events" fell into the study period

Indicate if critical "secular events" fell into the study period, e.g., significant changes in Internet resources available or "changes in computer hardware or Internet delivery resources"

	1	2	3	4	5	
subitem not at all important	○	○	○	○	○	essential

Does your paper address subitem 14a-i?

Copy and paste relevant sections from the manuscript (include quotes in quotation marks "like this" to indicate direct quotes from your manuscript), or elaborate on this item by providing additional information not in the ms, or briefly explain why the item is not applicable/relevant for your study

Your answer

14b) Why the trial ended or was stopped (early)

Does your paper address CONSORT subitem 14b? *

Copy and paste relevant sections from the manuscript (include quotes in quotation marks "like this" to indicate direct quotes from your manuscript), or elaborate on this item by providing additional information not in the ms, or briefly explain why the item is not applicable/relevant for your study

The trial did not end / was not stopped early.

15) A table showing baseline demographic and clinical characteristics for each group

NPT: When applicable, a description of care providers (case volume, qualification, expertise, etc.) and centers (volume) in each group

Does your paper address CONSORT subitem 15? *

Copy and paste relevant sections from the manuscript (include quotes in quotation marks "like this" to indicate direct quotes from your manuscript), or elaborate on this item by providing additional information not in the ms, or briefly explain why the item is not applicable/relevant for your study

"Table 2 summarizes the baseline characteristics. Among the 943 participants who consented and were randomized in the trial (bCBT=476; TAU=467) (See Figure S1 in Multimedia Appendix 1 for the trial’s profile), most were female (644/943, 68.3%), were middle-aged, and had a university degree or higher (447/943, 47.4). The PHQ-9 scores (median 15, IQR 7) reflected depression of moderate severity. PHQ-9 scores at 3 months will be reported in the main trial paper, which is being prepared. The median WAI-SR-C score was 47.42 (IQR 6) in the bCBT arm and 42 (IQR 8) in the TAU arm. The median SUS-C score was 42 (IQR 9) in the bCBT arm. See Table 3 for the median (IQR) values of the WAI-SR-C and SUS-C scores across treatment groups, and see Tables S6-S8 in Multimedia Appendix 1 for the median (IQR) values of the WAI-SR-C and SUS-C scores by country site."

15-i) Report demographics associated with digital divide issues

In ehealth trials it is particularly important to report demographics associated with digital divide issues, such as age, education, gender, social-economic status, computer/Internet/ehealth literacy of the participants, if known.

	1	2	3	4	5	
subitem not at all important	○	○	○	○	○	essential

Does your paper address subitem 15-i? *

Copy and paste relevant sections from the manuscript (include quotes in quotation marks "like this" to indicate direct quotes from your manuscript), or elaborate on this item by providing additional information not in the ms, or briefly explain why the item is not applicable/relevant for your study

Digital divide issues were not addressed in this paper.

16) For each group, number of participants (denominator) included in each analysis and whether the analysis was by original assigned groups

16-i) Report multiple "denominators" and provide definitions

Report multiple "denominators" and provide definitions: Report N's (and effect sizes) "across a range of study participation [and use] thresholds" [1], e.g., N exposed, N consented, N used more than x times, N used more than y weeks, N participants "used" the intervention/comparator at specific pre-defined time points of interest (in absolute and relative numbers per group). Always clearly define "use" of the intervention.

	1	2	3	4	5	
subitem not at all important	○	○	○	○	○	essential

Does your paper address subitem 16-i? *

Copy and paste relevant sections from the manuscript (include quotes in quotation marks "like this" to indicate direct quotes from your manuscript), or elaborate on this item by providing additional information not in the ms, or briefly explain why the item is not applicable/relevant for your study

Reports of multiple denominators were not used in the main analysis

16-ii) Primary analysis should be intent-to-treat

Primary analysis should be intent-to-treat, secondary analyses could include comparing only "users", with the appropriate caveats that this is no longer a randomized sample (see 18-i).

	1	2	3	4	5	
subitem not at all important	○	○	○	○	○	essential

Does your paper address subitem 16-ii?

Copy and paste relevant sections from the manuscript (include quotes in quotation marks "like this" to indicate direct quotes from your manuscript), or elaborate on this item by providing additional information not in the ms, or briefly explain why the item is not applicable/relevant for your study

Your answer

17a) For each primary and secondary outcome, results for each group, and the estimated effect size and its precision (such as 95% confidence interval)

Does your paper address CONSORT subitem 17a? *

Copy and paste relevant sections from the manuscript (include quotes in quotation marks "like this" to indicate direct quotes from your manuscript), or elaborate on this item by providing additional information not in the ms, or briefly explain why the item is not applicable/relevant for your study

"bCBT was associated with higher composite WAI-SR-C scores compared to TAU (B=5.67, 95% CI 4.48-6.86). Higher WAI-SR-C composite scores were associated with a significant decrease in PHQ-9 scores in bCBT (B=−0.12, 95% CI −0.17 to −0.06) and TAU (B=−0.06, 95% CI −0.11 to −0.02). Finally, there was a significant interaction between SUS-C and WAI SR-C with regard to an inverse association between higher WAI-SR-C scores and lower PHQ-9 scores in bCBT (b=−0.030, 95% CI −0.05 to −0.01; P=.005)."

Please see Tables 3, 4, 5 and Figure 1 for the full results.

17a-i) Presentation of process outcomes such as metrics of use and intensity of use

In addition to primary/secondary (clinical) outcomes, the presentation of process outcomes such as metrics of use and intensity of use (dose, exposure) and their operational definitions is critical. This does not only refer to metrics of attrition (13-b) (often a binary variable), but also to more continuous exposure metrics such as “average session length”. These must be accompanied by a technical description how a metric like a “session” is defined (e.g., timeout after idle time) [1] (report under item 6a).

	1	2	3	4	5	
subitem not at all important	○	○	○	○	○	essential

Does your paper address subitem 17a-i?

Copy and paste relevant sections from the manuscript (include quotes in quotation marks "like this" to indicate direct quotes from your manuscript), or elaborate on this item by providing additional information not in the ms, or briefly explain why the item is not applicable/relevant for your study

Your answer

17b) For binary outcomes, presentation of both absolute and relative effect sizes is recommended

Does your paper address CONSORT subitem 17b? *

Copy and paste relevant sections from the manuscript (include quotes in quotation marks "like this" to indicate direct quotes from your manuscript), or elaborate on this item by providing additional information not in the ms, or briefly explain why the item is not applicable/relevant for your study

This is not applicable to this study.

18) Results of any other analyses performed, including subgroup analyses and adjusted analyses, distinguishing pre-specified from exploratory

Does your paper address CONSORT subitem 18? *

Copy and paste relevant sections from the manuscript (include quotes in quotation marks "like this" to indicate direct quotes from your manuscript), or elaborate on this item by providing additional information not in the ms, or briefly explain why the item is not applicable/relevant for your study

"The sensitivity analysis with the complete case data set and subgroup analysis of 5 country sites that only offered face-to-face CBT in the TAU arm produced results that were comparable to those reported in the main paper. However, the interaction between SUS-C (and all subscales) and WAI-SR-C scores with regard to the association between WAI-SR-C and PHQ-9 scores was not significant in terms of sensitivity. Other differences are summarized in Results S2 in Multimedia Appendix 1, while the full results of the sensitivity and subgroup analyses can be found in Results S3 and S4 in Multimedia Appendix 1."

The full results, including estimates, of the sensitivity and subgroup analysis can be found in Appendix 1.

18-i) Subgroup analysis of comparing only users

A subgroup analysis of comparing only users is not uncommon in ehealth trials, but if done, it must be stressed that this is a self-selected sample and no longer an unbiased sample from a randomized trial (see 16-iii).

	1	2	3	4	5	
subitem not at all important	○	○	○	○	○	essential

Does your paper address subitem 18-i?

Copy and paste relevant sections from the manuscript (include quotes in quotation marks "like this" to indicate direct quotes from your manuscript), or elaborate on this item by providing additional information not in the ms, or briefly explain why the item is not applicable/relevant for your study

Your answer

19) All important harms or unintended effects in each group
(for specific guidance see CONSORT for harms)

Does your paper address CONSORT subitem 19? *

Copy and paste relevant sections from the manuscript (include quotes in quotation marks "like this" to indicate direct quotes from your manuscript), or elaborate on this item by providing additional information not in the ms, or briefly explain why the item is not applicable/relevant for your study

Based on the secondary analysis using working alliance data, no important harms or unintended effects were observed across treatment conditions.

19-i) Include privacy breaches, technical problems

Include privacy breaches, technical problems. This does not only include physical “harm” to participants, but also incidents such as perceived or real privacy breaches [1], technical problems, and other unexpected/unintended incidents. “Unintended effects” also includes unintended positive effects [2].

	1	2	3	4	5	
subitem not at all important	○	○	○	○	○	essential

Does your paper address subitem 19-i?

Copy and paste relevant sections from the manuscript (include quotes in quotation marks "like this" to indicate direct quotes from your manuscript), or elaborate on this item by providing additional information not in the ms, or briefly explain why the item is not applicable/relevant for your study

Your answer

19-ii) Include qualitative feedback from participants or observations from staff/researchers

Include qualitative feedback from participants or observations from staff/researchers, if available, on strengths and shortcomings of the application, especially if they point to unintended/unexpected effects or uses. This includes (if available) reasons for why people did or did not use the application as intended by the developers.

	1	2	3	4	5	
subitem not at all important	○	○	○	○	○	essential

Does your paper address subitem 19-ii?

Copy and paste relevant sections from the manuscript (include quotes in quotation marks "like this" to indicate direct quotes from your manuscript), or elaborate on this item by providing additional information not in the ms, or briefly explain why the item is not applicable/relevant for your study

Your answer

DISCUSSION

22) Interpretation consistent with results, balancing benefits and harms, and considering other relevant evidence

NPT: In addition, take into account the choice of the comparator, lack of or partial blinding, and unequal expertise of care providers or centers in each group

22-i) Restate study questions and summarize the answers suggested by the data, starting with primary outcomes and process outcomes (use)

Restate study questions and summarize the answers suggested by the data, starting with primary outcomes and process outcomes (use).

	1	2	3	4	5	
subitem not at all important	○	○	○	○	○	essential

Does your paper address subitem 22-i? *

Copy and paste relevant sections from the manuscript (include quotes in quotation marks "like this" to indicate direct quotes from your manuscript), or elaborate on this item by providing additional information not in the ms, or briefly explain why the item is not applicable/relevant for your study

"This study investigated the client-rated working alliance in a bCBT intervention for depression when compared to TAU [35]. Overall, our study found that treatment allocation (bCBT versus TAU) was a significant predictor of working alliance scores, in which ratings of the working alliance (composite scale and goals, task, and bond subscales) were higher in bCBT than in TAU. The working alliance was significantly associated with treatment outcomes. Across both bCBT and TAU groups, as working alliance scores increased, PHQ-9 scores decreased for composite, goals, and task scores but not for bond scores. Finally, there was a significant interaction between average and above-average system usability and higher working alliance (composite scale and goals and task subscales, but not bond subscale) scores when examining the relationship between the working alliance and PHQ-9 scores at 3-month assessments."

"Although our findings remain preliminary and do not show a causal effect, exploring the effect of the digital program on the working alliance may be a fruitful direction for future research."

22-ii) Highlight unanswered new questions, suggest future research

Highlight unanswered new questions, suggest future research.

	1	2	3	4	5	
subitem not at all important	○	○	○	○	○	essential

Does your paper address subitem 22-ii?

Copy and paste relevant sections from the manuscript (include quotes in quotation marks "like this" to indicate direct quotes from your manuscript), or elaborate on this item by providing additional information not in the ms, or briefly explain why the item is not applicable/relevant for your study

Your answer

20) Trial limitations, addressing sources of potential bias, imprecision, and, if relevant, multiplicity of analyses

20-i) Typical limitations in ehealth trials

Typical limitations in ehealth trials: Participants in ehealth trials are rarely blinded. Ehealth trials often look at a multiplicity of outcomes, increasing risk for a Type I error. Discuss biases due to non-use of the intervention/usability issues, biases through informed consent procedures, unexpected events.

	1	2	3	4	5	
subitem not at all important	○	○	○	○	○	essential

Does your paper address subitem 20-i? *

Copy and paste relevant sections from the manuscript (include quotes in quotation marks "like this" to indicate direct quotes from your manuscript), or elaborate on this item by providing additional information not in the ms, or briefly explain why the item is not applicable/relevant for your study

"Several study limitations should be noted. First, working alliance data were collected at a single point that corresponded with 3-month assessments. While this is common in clinical trials [25,58], the measurement of the alliance is recommended early in treatment within the first 5 sessions and at different points across treatment [74-77]. However, the number of face-to-face sessions varied between the 9 country sites (eg, 5 to 10 sessions), which would have posed significant challenges for the systematic data collection required in a clinical trial [54]. Second, the study engaged in multiple comparisons, which may have increased the risk of type 1 error (a positive result may be due to chance). However, given the exploratory nature of this analysis and the fact that different outcomes are likely to be highly correlated, a multiple adjustment comparison was not deemed necessary [78]. Third, the results of the analysis are valid under the MAR assumption, which we believe to be plausible because the effect of country sites appears to influence the missingness of the main outcome variables, stemming from country-specific data collection procedures and experiences. This is supported by chi-square analyses that indicate significantly higher rates of missing data for the PHQ-9 and WAI-SR-C across some countries compared to others. Nevertheless, it should be noted that this paper cannot rule out that data are missing not at random. Future research can explore this further using a sensitivity analysis. Fourth, the heterogeneity of interventions offered in the TAU group limits the study from conclusively tying causation to a specific comparator intervention. However, it should be noted that interventions offered by services in TAU were regarded as evidence-based, largely consisting of CBT and psychopharmacological interventions [35]. This may reduce the limitations associated with the multiple treatments offered in TAU [66,79] and adhering to the pragmatic trial's ancillary objective to not impose specific constraints on clients and clinicians concerning data collection [79]. However, additional steps were also taken to address this limitation by conducting a subanalysis with a subset of trial country sites that only offered face-to-face CBT in TAU. The findings showed comparable results to those of the main analysis, highlighting that the addition of iCBT to face-to-face CBT may improve the quality of the working alliance. Fifth, another potential limitation is related to the variation in how bCBT was delivered across the trial's country sites, concerning the number of sessions offered across each site and the types of iCBT programs delivered. The bCBT arm was focused on investigating the noninferiority of bCBT given that there is a sufficient level of evidence concerning other treatment components, such as the CBT approach, and different delivery formats, including in-person delivery and internet-based mental health interventions for depression [80,81]. Although the number of treatment sessions varied between settings, to our knowledge, there is no evidence to suggest that the number of sessions of CBT effect the client-therapist alliance as the alliance is typically developed early in treatment and within the first 5 sessions [74-77]. Moreover, another study exploring the usage of different components of bCBT and treatment engagement when compared to intended use in the E-COMPARED study concluded that personalized blended care was more suitable compared to attempting to achieve a standardized optimal blend [69]. Variations in the number of treatment sessions described may enable a pragmatic understanding of the working alliance in interventions in real-world clinical settings [66]."

21) Generalisability (external validity, applicability) of the trial findings

NPT: External validity of the trial findings according to the intervention, comparators, patients, and care providers or centers involved in the trial

21-i) Generalizability to other populations

Generalizability to other populations: In particular, discuss generalizability to a general Internet population, outside of a RCT setting, and general patient population, including applicability of the study results for other organizations

	1	2	3	4	5	
subitem not at all important	○	○	○	○	○	essential

Does your paper address subitem 21-i?

Copy and paste relevant sections from the manuscript (include quotes in quotation marks "like this" to indicate direct quotes from your manuscript), or elaborate on this item by providing additional information not in the ms, or briefly explain why the item is not applicable/relevant for your study

Your answer

21-ii) Discuss if there were elements in the RCT that would be different in a routine application setting

Discuss if there were elements in the RCT that would be different in a routine application setting (e.g., prompts/reminders, more human involvement, training sessions or other co-interventions) and what impact the omission of these elements could have on use, adoption, or outcomes if the intervention is applied outside of a RCT setting.

	1	2	3	4	5	
subitem not at all important	○	○	○	○	○	essential

Does your paper address subitem 21-ii?

Copy and paste relevant sections from the manuscript (include quotes in quotation marks "like this" to indicate direct quotes from your manuscript), or elaborate on this item by providing additional information not in the ms, or briefly explain why the item is not applicable/relevant for your study

Your answer

OTHER INFORMATION

23) Registration number and name of trial registry

Does your paper address CONSORT subitem 23? *

Copy and paste relevant sections from the manuscript (include quotes in quotation marks "like this" to indicate direct quotes from your manuscript), or elaborate on this item by providing additional information not in the ms, or briefly explain why the item is not applicable/relevant for your study

"France: ClinicalTrials.gov NCT02542891, September 4, 2015; Germany: German Clinical Trials Register DRKS00006866, December 2, 2014; The Netherlands: Netherlands Trials Register NTR4962, January 5, 2015; Poland: ClinicalTrials.gov NCT02389660, February 18, 2015; Spain: ClinicalTrials.gov NCT02361684, January 8, 2015; Sweden: ClinicalTrials.gov NCT02449447, March 30, 2015; Switzerland: ClinicalTrials.gov NCT02410616, April 2, 2015; United Kingdom: ISRCTN Registry ISRCTN12388725, March 20, 2015; Denmark: ClinicalTrials.gov NCT02796573, June 1, 2016) [35,38]"

24) Where the full trial protocol can be accessed, if available

Does your paper address CONSORT subitem 24? *

Cite a Multimedia Appendix, other reference, or copy and paste relevant sections from the manuscript (include quotes in quotation marks "like this" to indicate direct quotes from your manuscript), or elaborate on this item by providing additional information not in the ms, or briefly explain why the item is not applicable/relevant for your study

Published E-COMPARED Protocol:
Kleiboer, A., Smit, J., Bosmans, J. et al. European COMPARative Effectiveness research on blended Depression treatment versus treatment-as-usual (E-COMPARED): study protocol for a randomized controlled, non-inferiority trial in eight European countries. Trials 17, 387 (2016). https://doi.org/10.1186/s13063-016-1511-1

25) Sources of funding and other support (such as supply of drugs), role of funders

Does your paper address CONSORT subitem 25? *

Copy and paste relevant sections from the manuscript (include quotes in quotation marks "like this" to indicate direct quotes from your manuscript), or elaborate on this item by providing additional information not in the ms, or briefly explain why the item is not applicable/relevant for your study

The E-COMPARED trial was funded by the European Commission FP7-Health-2013-Innovation-1 program (grant agreement number: 603098)

X27) Conflicts of Interest (not a CONSORT item)

X27-i) State the relation of the study team towards the system being evaluated

In addition to the usual declaration of interests (financial or otherwise), also state the relation of the study team towards the system being evaluated, i.e., state if the authors/evaluators are distinct from or identical with the developers/sponsors of the intervention.

	1	2	3	4	5	
subitem not at all important	○	○	○	○	○	essential

Does your paper address subitem X27-i?

Copy and paste relevant sections from the manuscript (include quotes in quotation marks "like this" to indicate direct quotes from your manuscript), or elaborate on this item by providing additional information not in the ms, or briefly explain why the item is not applicable/relevant for your study

Your answer

About the CONSORT EHEALTH checklist

As a result of using this checklist, did you make changes in your manuscript? *

- ○ yes, major changes
- ◉ yes, minor changes
- ○ no

What were the most important changes you made as a result of using this checklist?

Your answer

How much time did you spend on going through the checklist INCLUDING making changes in your manuscript *

It took between 1-2 hours to complete.

As a result of using this checklist, do you think your manuscript has improved? *

- [x] yes
- [ ] no
- [ ] Other:

Would you like to become involved in the CONSORT EHEALTH group?

This would involve for example becoming involved in participating in a workshop and writing an "Explanation and Elaboration" document

- [ ] yes
- [x] no
- [ ] Other:

Clear selection

Any other comments or questions on CONSORT EHEALTH

Your answer

STOP - Save this form as PDF before you click submit

To generate a record that you filled in this form, we recommend to generate a PDF of this page (on a Mac, simply select "print" and then select "print as PDF") before you submit it.

When you submit your (revised) paper to JMIR, please upload the PDF as supplementary file.

Don't worry if some text in the textboxes is cut off, as we still have the complete information in our database. Thank you!

Final step: Click submit !

Click submit so we have your answers in our database!

Submit

Never submit passwords through Google Forms.

This content is neither created nor endorsed by Google. Report Abuse - Terms of Service - Privacy Policy

Google Forms
